# LncRNA MRF drives the regulatory function on monocyte recruitment and polarization through HNRNPD-MCP1 axis in mesenchymal stem cells

**DOI:** 10.1186/s12929-022-00858-3

**Published:** 2022-09-21

**Authors:** Jiajie Lin, Zhongyu Xie, Zhaoqiang Zhang, Ming Li, Guiwen Ye, Wenhui Yu, Jinteng Li, Feng Ye, Zepeng Su, Yunshu Che, Peitao Xu, Chenying Zeng, Peng Wang, Yanfeng Wu, Huiyong Shen

**Affiliations:** 1 Department of Orthopedics, The Eighth Affiliated Hospital, Sun Yat-Sen University, Shenzhen, 518000 China; 2 Center for Biotherapy, The Eighth Affiliated Hospital, Sun Yat-Sen University, Shenzhen, 518000 China; 3 Department of Orthopedics, Sun Yat-Sen Memorial Hospital, Sun Yat-Sen University, Guangzhou, 510000 China

**Keywords:** Long noncoding RNAs, Mesenchymal stem cells, Immunomodulation, MRF, MCP1

## Abstract

**Background:**

Mesenchymal stem cells (MSCs) exhibit two bidirectional immunomodulatory abilities: proinflammatory and anti-inflammatory regulatory effects. Long noncoding RNAs (lncRNAs) have important functions in the immune system. Previously, we performed high-throughput sequencing comparing lncRNA expression profiles between MSCs cocultured with or without CD14+ monocytes and screened out a new lncRNA termed lncRNA MCP1 regulatory factor (MRF). However, the mechanism of MRF in MSCs is still unknown.

**Methods:**

MRF expression was quantified via qRT–PCR. RNA interference and lentiviruses were used to regulate MRF expression. The immunomodulatory effects of MSCs on monocytes were evaluated via monocyte migration and macrophage polarization assays. RNA pull-down and mass spectrometry were utilized to identify downstream factors of MRF. A dual-luciferase reporter assay was applied to analyze the transcription factors regulating MRF. qRT–PCR, western blotting and ELISAs were used to assess MCP1 expression. A human monocyte adoptive transfer mouse model was applied to verify the function of MRF in vivo.

**Results:**

MRF was upregulated in MSCs during coculture with CD14+ monocytes. MRF increased monocyte recruitment by upregulating the expression of monocyte chemotactic protein (MCP1). Knockdown of MRF enhanced the regulatory effect of MSCs on restraining M1 polarization and facilitating M2 polarization. Mechanistically, MRF bound to the downstream protein heterogeneous nuclear ribonucleoprotein D (HNRNPD) to upregulate MCP1 expression, and the transcription factor interferon regulatory factor 1 (IRF1) activated MRF transcription early during coculture. The human monocyte adoptive transfer model showed that MRF downregulation in MSCs inhibited monocyte chemotaxis and enhanced the effects of MSCs to inhibit M1 macrophage polarization and promote M2 polarization in vivo.

**Conclusion:**

We identified the new lncRNA MRF, which exhibits proinflammatory characteristics. MRF regulates the ability of MSCs to accelerate monocyte recruitment and modulate macrophage polarization through the HNRNPD-MCP1 axis and initiates the proinflammatory regulatory process in MSCs, suggesting that MRF is a potential target to improve the clinical effect of MSC-based therapy or correct MSC-related immunomodulatory dysfunction under pathological conditions.

**Supplementary Information:**

The online version contains supplementary material available at 10.1186/s12929-022-00858-3.

## Background

Mesenchymal stem cells (MSCs) are a type of adult stem cell that can be derived from various tissue types, such as bone marrow, umbilical cord blood, and fat. MSCs exhibit self-renewal, trilineage differentiation potential, and immunomodulation [1]. MSCs can mediate broad-spectrum immunomodulation and generally modulate immunologic processes in many innate and adaptive immune cells, influencing cell development, differentiation and activation to maintain homeostasis of the microenvironment [2, 3]. Due to the powerful immunomodulatory capacity of MSCs, these cells have recently been used in many clinical studies and trials to suppress inflammation and treat diseases, particularly autoimmune diseases, such as multiple sclerosis, graft-versus-host disease (GVHD) and atopic dermatitis [4]. According to recent studies, MSCs exhibit bidirectional immunomodulation capacities under different circumstances [2, 5, 6]. When the immune system is not activated, MSCs exert proinflammatory functions in response to external stimulation. Once the immune system is activated, MSCs will transform into an immunosuppressive state to prevent overactivation of the immune response. However, the specific mechanisms by which MSCs exert immunomodulatory functions remain to be elucidated.

CD14+ monocytes are myeloid cells derived from the bone marrow that differentiate into macrophages and dendritic cells [7]. In the context of microbial infection or injury, circulating blood monocytes migrate into tissues and further mature, secrete cytokines, engulf pathogens and clear cellular debris [8]. However, abnormal monocyte recruitment induces pathological inflammation, which has been observed in individuals with atherosclerosis and rheumatoid arthritis (RA) [9]. The migration of monocytes is mainly driven by monocyte chemotactic protein 1 (MCP1, also known as CC-chemokine ligand 2 (CCL2)) [10]. MSCs are one of the major sources of secreted MCP1 [11]. Previously, we confirmed that MSCs cocultured with monocytes were induced to express more MCP1, which subsequently recruited monocytes [12]. Furthermore, we reported that MSCs derived from patients with ankylosing spondylitis (AS) overexpressed MCP1 and excessively promoted monocyte migration, leading to monocyte dysfunction and chronic inflammation in AS [13]. How MSCs regulate MCP1 expression when cocultured with monocytes is still unknown.

Long noncoding RNAs (lncRNAs) are RNA transcripts containing more than 200 nucleotides that lack protein-coding potential [14]. More lncRNA genes than protein-coding genes are present in the human genome, but most are unidentified [15]. In recent years, lncRNAs have been shown to play important roles in MSC trilineage differentiation [16–18], but few studies have investigated whether lncRNAs participate in MSC-mediated immunomodulation. Previously, we performed high-throughput sequencing to compare lncRNA expression profiles between MSCs cocultured with or without CD14+ monocytes [12]. A total of 145 lncRNAs exhibited significantly different expression levels, indicating that these lncRNAs might mediate the immunomodulatory effect of MSCs on monocytes, but the concrete functions and mechanisms remain unclear.

In this study, we identified a new lncRNA in MSCs termed lncRNA MCP1 regulatory factor (MRF). MRF exhibited powerful proinflammatory characteristics that led to recruitment of monocytes. Knockdown of MRF enhanced the regulatory effect of MSCs on restraining M1 polarization and facilitating M2 polarization. Mechanistically, MRF increased the level of MCP1 by binding to heterogeneous nuclear ribonucleoprotein D (HNRNPD), and MRF was upregulated by the transcription factor interferon regulatory factor 1 (IRF1) during coculture with monocytes. Our findings support the notion that MRF might activate a proinflammatory response in MSCs and that MRF is a potential target to improve the clinical efficacy of MSC-based therapy or correct MSC-related immunomodulatory dysfunction under pathological conditions.

## Methods

### Cell isolation and culture

Bone marrow was extracted from the posterior superior iliac spine of healthy volunteers under sterile conditions. Then, bone marrow-derived mesenchymal stem cells (BMMSCs) were isolated and purified by density gradient centrifugation as previously reported [12]. BMMSCs at passage two were used in experiments and cultured in medium composed of 90% Dulbecco’s modified Eagle’s medium (DMEM, Gibco, New York, USA) and 10% fetal bovine serum (FBS, TIANHANG, Zhejiang, China) at 37 °C in a 5% CO_2_ atmosphere. The culture medium was replaced every third day. Peripheral blood mononuclear cells (PBMCs) were isolated via density gradient centrifugation. CD14+ monocytes were isolated from PBMCs with CD14 MicroBeads (Miltenyi Biotec, Bergisch Gladbach, Germany).

### Monocyte migration assay

Polycarbonate Membrane Transwell® Inserts (5-µm pores, Corning, New York, USA) were used to perform monocyte migration experiments. MSCs (2 × 10^4^) or cell-free MSC culture supernatants were placed in the lower chambers, and CD14+ monocytes (1 × 10^6^) were seeded in the upper chambers. Both sets of cells were cultured in RPMI 1640 medium supplemented with 10% FBS at 37 ℃ in a 5% CO_2_ atmosphere. Twelve hours later, the culture supernatants in the lower chambers were collected, and the number of CD14+ monocytes was determined via flow cytometry.

### Macrophage polarization assay

CD14+ monocytes were cocultured with MSCs using Polycarbonate Membrane Transwell® Inserts (0.4-µm pores). CD14+ monocytes (2 × 10^5^) were seeded in the lower chambers, and MSCs (2 × 10^4^) were seeded in the upper chambers. Recombinant human M-CSF (25 ng/ml, PeproTech, New Jersey, USA) was added to activate monocytes. Five days later, lipopolysaccharide (LPS; 50 ng/ml, SigmaAldrich, Darmstadt, Germany) and recombinant human interferon-γ (IFN-γ; 20 ng/ml, PeproTech) were added to induce macrophage polarization to the M1 phenotype, and recombinant human IL-4 (20 ng/ml, PeproTech) and IL-10 (20 ng/ml, PeproTech) were added to induce macrophage polarization to the M2 phenotype. Twenty-four hours later, the phenotype of the macrophages was determined by flow cytometry. Macrophages were digested, incubated with anti-HLA-DR-PE antibody or anti-CD206-BV421 antibody (BD Biosciences, California, USA) and then incubated with fixation medium (Invitrogen, Massachusetts, USA) for 15 min. After three washes with PBS, the cells were incubated with permeabilization medium plus an anti-CD68-FITC antibody (BD Biosciences) for 30 min.

### Rapid amplification of cDNA ends (RACE)

A SMARTer RACE amplification kit (Clontech, California, USA) was used to perform RACE. In brief, total RNA was extracted from MSCs and used to synthesize first-strand cDNA for 3′- and 5′-RACE. Then, 5′-RACE and 3′-RACE PCRs were performed using specific primers (Additional file 1: Table S1), and 3′- and 5′ nested PCRs were performed using products from the aforementioned PCRs as templates. DNA gel electrophoresis was used to identify the target fragments. These fragments were collected and cloned into a plasmid, and the full-length cDNAs were subsequently evaluated via sequencing.

### RNA interference

siRNAs specific for MRF, IRF1, HNRNPD and a negative control siRNA were generated by IGE (Guangzhou, China). MSCs were transfected with siRNAs using Opti-MEM (GIBCO) and Lipofectamine RNAiMAX (Thermo Fisher, Massachusetts, USA) according to the manufacturer’s directions. Knockdown efficiency was analyzed via qRT–PCR or western blotting after 72 h.

### Lentiviral assay

A full-length MRF-overexpression lentivirus and a negative control were generated by OBiO (Shanghai, China). MSCs were infected with the MRF-overexpression lentivirus or vector (multiplicity of infection: 50) using 5 µg/ml polybrene. The medium containing the lentivirus was replaced with fresh medium 24 h later. Overexpression efficiency was analyzed via qRT–PCR after 96 h.

### Dual-luciferase reporter assay

The promoter sequence of MRF from − 2000 to + 100 bp relative to the transcription start site and the antisense sequence were synthesized and separately cloned into a pGL4.10 vector. 293 T cells were transfected with the above vectors followed by transfection with siRNAs to silence IRF1 using Lipofectamine 3000 (Invitrogen). All experimental group cells were transfected with pRL-TK plasmids as an internal control. Luciferase activities were detected with a Dual-Luciferase Reporter Assay System (Promega, E1910, Massachusetts, USA). The relative luciferase intensity is shown as the numerical value of firefly luciferase activity divided by Renilla luciferase activity.

### RNA pull-down and mass spectrometry

In vitro transcription of MRF and the antisense sequence was performed with a TranscriptAid T7 High Yield Transcription Kit (Thermo, K0441). The RNA products were purified and subsequently biotinylated using a Pierce RNA 3′ End Desthiobiotinylation Kit (Thermo, 20163). MSC protein lysates were prepared using standard IP lysis buffer with protease inhibitor. RNA pull-down was performed using a Pierce Magnetic RNA‒Protein Pull-Down Kit (Thermo, 20164) according to the manufacturer’s instructions. Briefly, the biotinylated RNA was captured with streptavidin magnetic beads, and the labeled RNA-magnetic bead complexes were then incubated with MSC protein lysates for 60 min. The protein-biotinylated RNA-magnetic bead complexes were collected with a magnetic stand, and RNA-binding proteins were washed with buffer and finally eluted for subsequent analysis. Silver staining was used to detect distinct protein bands, and further identification was performed using mass spectrometry analysis with an LC–MS/MS system (Thermo Scientific Q Exactive).

### Cell cytoplasmic and nuclear fractionation

A PARIS kit (Thermo Fisher) was used to separate the cell cytoplasmic and nuclear fractions. The RNA in each fraction was extracted and reverse transcribed into cDNA. The distribution of target mRNA in each fraction was analyzed via qRT–PCR. U6 and MALAT1 were used as positive controls for the nuclear fraction, while GAPDH and ACTIN were used as positive controls for the cytoplasmic fraction.

### RNA extraction and quantitative real-time PCR

To extract total RNA from MSCs, first, MSCs were washed 3 times with phosphate-buffered saline, TRIzol (TaKaRa, Dalian, China) was added to lyse the cells, and extraction was performed according to the manufacturer’s instructions. Specifically, to extract total RNA from MSCs cocultured with monocytes to assess the RNA level of MCP1 and eliminate interference from MCP1 derived from monocytes, a transwell system was used to perform coculture. MSCs (2 × 10^4^) were seeded in the lower chambers, and CD14+ monocytes (1 × 10^6^) were cultured in the upper chambers. Then, the upper chambers containing monocytes and supernatants were removed, MSCs in the lower chambers were washed 3 times with phosphate-buffered saline, and TRIzol (TaKaRa) was added to lyse the cells. Total RNA was reverse transcribed into cDNA with a PrimeScript PT Reagent Kit (TaKaRa). Quantitative real-time PCR was performed using a SYBR Premix Ex Taq Kit (TaKaRa). The PCR procedure was set as follows: 95 °C for 1 min, 40 cycles at 95 °C for 30 s, 58 °C for 20 s, and 72 °C for 30 s, and finally 72 °C for 5 min for full elongation of the products. The results were analyzed using the 2^−ΔΔCt^ method, and GAPDH was considered the reference gene to calculate relative gene expression. The forward and reverse primers are listed in Additional file 1: Table S1.

### Western blotting analysis

To collect proteins, cell lysates were extracted with RIPA lysis buffer containing 1% protease and phosphatase inhibitors. Specifically, to detect the MCP1 protein level in MSCs cocultured with monocytes and eliminate interference from MCP1 derived from monocytes, coculture was performed as described above, MSCs in the lower chambers were washed 3 times with phosphate-buffered saline, and RIPA buffer was added to extract cell lysates. Then, the proteins were separated via sodium dodecyl sulfate–polyacrylamide gel electrophoresis (SDS–PAGE) and transferred to polyvinylidene fluoride membranes. The membranes were blocked and incubated with primary antibodies, followed by incubation with HRP-conjugated secondary antibodies. Protein levels were detected with a chemiluminescent HRP substrate (Millipore, Vermont, USA) and quantified using ImageJ. The following antibodies were used in this study: anti-MCP1 (Abcam, ab9669, Cambridge, UK), anti-IRF1 (Cell Signaling Technology, 8478S, Massachusetts, USA), anti-HNRNPD (Cell Signaling Technology, 12382S), anti-GAPDH (Cell Signaling Technology, 5174S) and anti-β-tubulin (Cell Signaling Technology, 2128S).

### ELISAs

To detect MCP1 secreted from MSCs cocultured with monocytes, coculture was performed as described above. After removal of the upper chambers and culture supernatants, fresh medium was added to the lower chambers, and MSCs were cultured for an additional 24 h to eliminate the interference from MCP1 produced by monocytes. Then, cell culture supernatants were collected and analyzed with a human MCP1 ELISA kit (R&D, DCP00, Minnesota, USA) according to the manufacturer’s instructions. Briefly, the supernatants and standard were added to a microplate and incubated for 2 h at room temperature. Then, the samples were aspirated and washed 3 times with washing buffer, and human MCP-1 conjugate was added to the microplate and incubated for 1 h at room temperature. After three washes, substrate and stop solutions were successively added to the wells. Optical density (O.D.) was measured with a microplate reader, and a standard curve was created using standard samples. Accurate MCP1 concentrations were calculated based on the standard curve.

### RNA sequencing

LncRNA and mRNA high-throughput sequencing was performed as previously described [12]. Briefly, MSCs (n = 5) cocultured with or without CD14+ monocytes were separately treated with TRIzol (TaKaRa). RNA was extracted according to the manufacturer’s protocol, and RNA integrity was evaluated with an Agilent 2200 TapeStation (Agilent Technologies, USA). Then, RNA was fragmented into average sizes of approximately 200 nt and reverse transcribed into single-stranded cDNAs. Double-stranded cDNAs were synthesized, purified and treated with terminal repair and ligation primers using the NEBNext® Ultra™ RNA Library Prep Kit for Illumina (NEB, USA). After PCR amplification and purification, libraries were paired-end sequenced (PE150) on an Illumina HiSeq 3000 platform at Guangzhou RiboBio Co., Ltd. (Guangzhou, China). Differential expression was analyzed using DESeq2 with |log2Fold Change|> 1 and Q value < 0.05.

### Bioinformatics analysis

A heatmap was drawn with pheatmap (v1.0.12) based on differentially expressed genes. An advanced volcano plot was generated using the OmicStudio tools (https://www.omicstudio.cn/tool) based on OmicStudioClassic (v1.3.14) and OmicStudioKit (v1.9.0). A coexpression-based GO enrichment analysis of lncRNAs was performed using the AnnoLnc2 database (http://annolnc.gao-lab.org/) [19], and significantly enriched GO terms with a Q value < 0.05 were reported as putative functional annotations of the lncRNAs. The GO enrichment analysis of differentially expressed genes was performed with the DAVID database (https://david.ncifcrf.gov/), and a P value < 0.05 was considered to indicate significant enrichment in the annotation categories. GO terms related to the immune response were selected to perform a GOChord analysis using the R package GOplot (v1.0.2). Potential transcription factors binding to the MRF promotor were predicted via the JASPAR database (https://jaspar.genereg.net/), and the top 30 predicted transcription factors were considered for subsequent screening.

### Monocyte recruitment and macrophage polarization in vivo

NOD/SCID mice were subcutaneously injected with 0.5 mg/kg recombinant human M-CSF (PeproTech, 300-25) before adoptive transfer [20, 21]. For monocyte recruitment, human MSCs pretreated with MRF siRNA or the negative control were intraperitoneally injected into the mice. Twelve hours later, 1 × 10^7^ human CD14+ monocytes labeled with CFSE were intravenously injected into each mouse. After 24 h, the adoptively transferred mice were sacrificed, and peritoneal lavage fluid and spleen cells were collected for flow cytometry analysis. For macrophage polarization, human MSCs were pretreated with MRF siRNA or negative control, human macrophages were activated by human M-CSF and then stimulated with LPS and recombinant human IFN-γ to induce M1 polarization or with recombinant human IL-4 and IL-10 to induce M2 polarization, similar to the in vitro induction of macrophage polarization. Then, 1 × 10^5^ human MSCs and 1 × 10^6^ macrophages labeled with CFSE were simultaneously injected into the abdominal cavity of NOD/SCID mice. Two days later, transplanted mice were sacrificed, and peritoneal lavage fluid was collected to detect the MFI of human HLA-DR and human CD206 in CFSE-labeled cells via flow cytometry.

### Statistical analysis

The data in this study were analyzed using SPSS 26.0. The results are presented as the mean ± standard deviation (SD). Independent-sample t tests were used to compare two experimental groups. One-way ANOVA with Bonferroni’s test was used to analyze differences among three or more groups. *P* < 0.05 was considered to indicate a significant difference.

## Results

### MRF is upregulated in MSCs cocultured with CD14+ monocytes

**Fig. 1:**
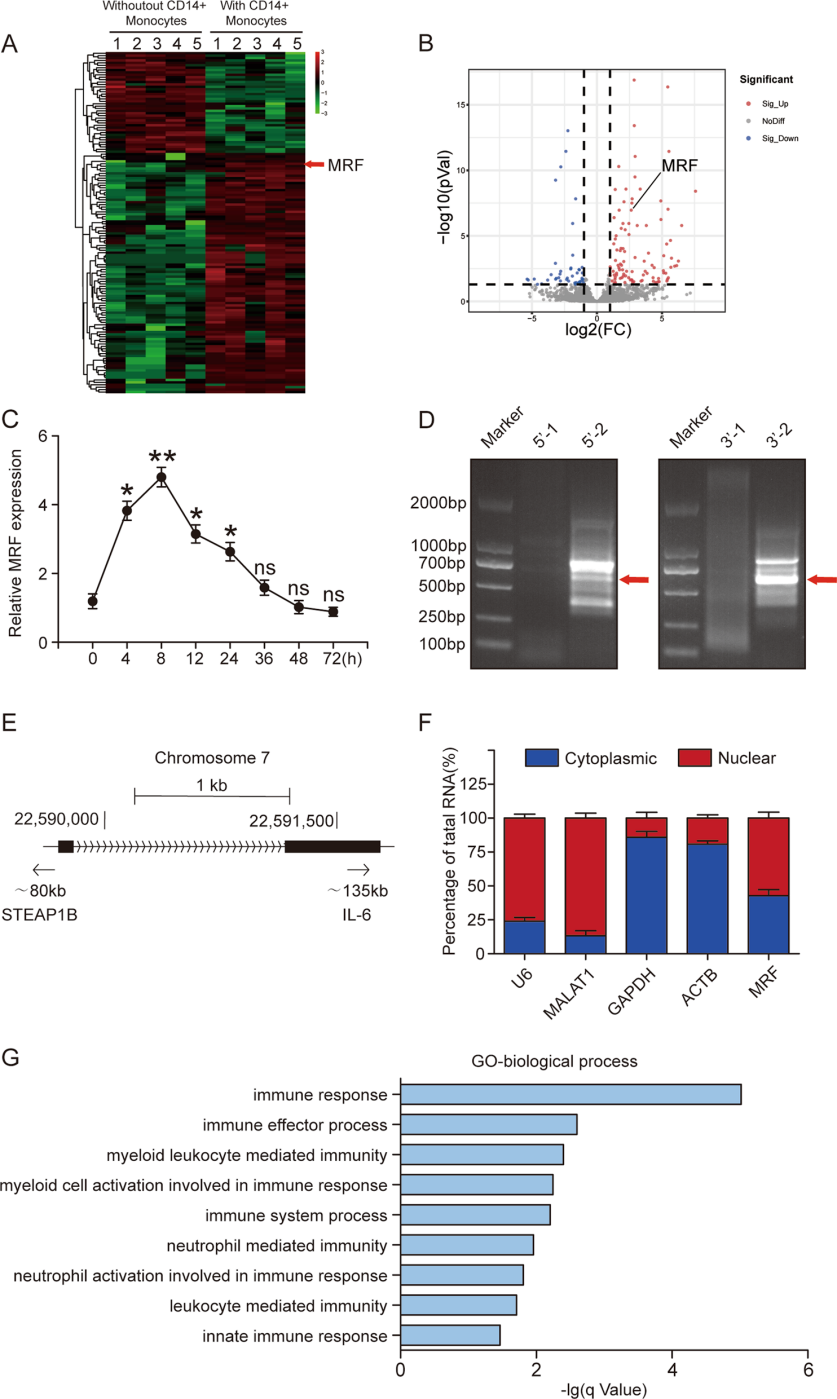
MRF is upregulated in MSCs during coculture with CD14+ monocytes. **A** Heatmap of differentially expressed lncRNAs (≥ two-fold) in MSCs between cells cocultured with or without CD14+ monocytes. **B** Volcano plots of lncRNA high-throughput sequencing results for MSCs compared between coculture with or without CD14+ monocytes. **C** Dynamic expression of MRF at different time points during coculture, as measured by qRT–PCR (n = 3). **D** DNA gel electrophoresis of RACE products: 5′-1,5′-RACE PCR; 5′-2,5′-RACE nested PCR; 3′-1,3′-RACE PCR; 3′-2,3′-RACE nested PCR. The red arrows indicate the correct fragments produced by RACE. **E** Schematic annotation of MRF in the genome. **F** Percentages of cytoplasmic and nuclear RNA in MSCs assessed by qRT–PCR after cytoplasmic and nuclear fractionation. U6 and MALAT1 represent nuclear RNA, and GAPDH and ACTB represent cytoplasmic RNA (n = 3). **G** GO analysis of genes coexpressed with MRF using the AnnoLnc2 database. The data are presented as the mean ± SD; *P < 0.05, **P < 0.01, *ns* not significant. All experiments were repeated independently three times

To investigate the relationship between lncRNAs and the immunomodulation of monocytes by MSCs, we previously performed high-throughput sequencing comparing the lncRNA expression profiles between MSCs cocultured with or without CD14+ monocytes [12] (Fig. 1A and B). We identified a new lncRNA, which we termed lncRNA MRF. MRF was obviously upregulated when MSCs were cocultured with CD14+ monocytes, peaking at 8 h and then gradually decreasing (Fig. 1C). To identify the characteristics of MRF, we performed a RACE assay to obtain the full-length lncRNA transcript (Fig. 1D). MRF was located on chromosome 7 in the human genome (Fig. 1E). Analysis of the protein-coding potential was conducted with three different algorithms: Coding Potential Assessment Tool (CPAT), Coding Potential Calculator (CPC) and PhyloCSF. All the results showed that MRF did not have the ability to encode a protein (Additional file 2: Fig. S1A). A conservation analysis using PhyloP and PhastCons indicated that MRF was a modestly conserved lncRNA (Additional file 2: Fig. S1B). Furthermore, MRF was located in both the cytoplasm and the nucleus in comparison to U6 RNA and lncRNA MALAT1 located in the nucleus or GAPDH and ACTB located in the cytoplasm (Fig. 1F). We performed a GO analysis based on genes coexpressed with MRF using the AnnoLnc2 database. The coexpressed genes were enriched in immune response-related processes, suggesting that MRF was related to the immunomodulatory function of MSCs (Fig. 1G). Overall, we identified a new immune-related lncRNA in MSCs named MRF, which was upregulated when MSCs were cocultured with monocytes.

### Knockdown of MRF inhibits monocyte recruitment and enhances the regulatory effect of MSCs on macrophage polarization

**Fig. 2:**
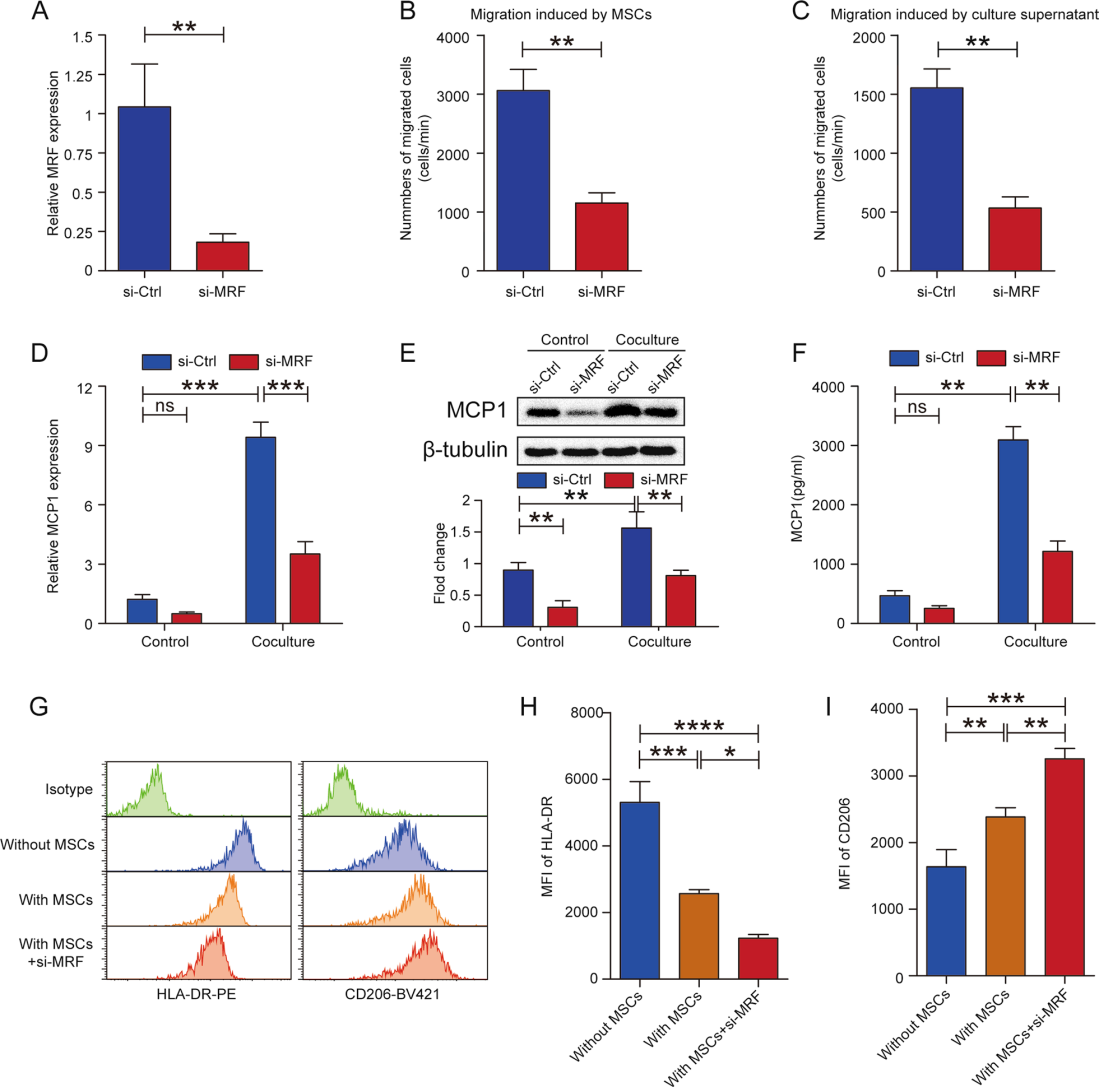
Knockdown of MRF in MSCs inhibits monocyte recruitment and M1 macrophage polarization but facilitates M2 polarization. **A** The downregulation of MRF expression was verified by qRT–PCR after knockdown (n = 3). **B** The number of migrated monocytes induced by MSCs was decreased after MRF knockdown (n = 3). **C** The number of migrated monocytes induced by MSC culture supernatant was decreased after MRF knockdown (n = 3). **D** The MCP1 mRNA level in MSCs was measured by qRT–PCR after MRF knockdown under the conditions of coculture with or without CD14+ monocytes (n = 3). **E** The MCP1 protein level in MSCs was detected by western blotting, and the quantitative results were normalized to the result for β-tubulin (n = 3). **F** MCP1 levels in MSC culture supernatants were quantified via ELISA (n = 3). **G** The MFI of HLA-DR (left panel) and CD206 (right panel) was detected in CD68-positive cells via flow cytometric analysis (n = 3). **H** Histogram showing the MFI of HLA-DR among the groups cultured without MSCs, with MSCs and with MRF-knockdown MSCs. **I** Histogram showing the MFI of CD206 among the groups cultured without MSCs, with MSCs and with MRF-knockdown MSCs. The data are presented as the mean ± SD; *P < 0.05, **P < 0.01, ***P < 0.001, ****P < 0.0001, *ns* not significant. All experiments were independently repeated three times

We previously demonstrated that MSCs can increase the migration of monocytes and inhibit M1 macrophage polarization; thus, we wondered whether MRF participates in these processes. We first performed knockdown of MRF in MSCs using small interfering RNAs (siRNAs), and the knockdown efficiency was verified by qRT–PCR (Fig. 2A). Migration assays showed that both MSCs and MSC culture supernatants recruited fewer monocytes upon MRF knockdown (Fig. 2B and C). Many studies have confirmed that MCP1 was one of the main chemokines derived from MSCs that mediated monocyte recruitment [11, 13, 22]. qRT–PCR, western blotting and ELISA results indicated that the expression level of MCP1 in MSCs was upregulated, peaked at 24 h, lasted for approximately 72 h and then gradually decreased after 120 h of coculture with monocytes (Additional file 3: Fig. S2A–S2C). In order to eliminate the interference from MCP1 produced by monocytes, we also performed knockdown of MCP1 in monocytes before coculture with MSCs. The knockdown efficiency was verified via qRT–PCR, western blotting and ELISA (Additional file 3: Fig. S2D–S2F), the dynamic changes of MCP1 expression in MSCs during coculture were assessed under such condition, and the results showed the similar trend (Additional file 3: Fig. S2G–S2I).

To confirm whether MCP1 was involved in MRF-mediated monocyte recruitment, we detected MCP1 expression in MSCs with MRF knockdown. The results showed that regardless of the coculture status, knockdown of MRF decreased MCP1 expression in MSCs (Fig. 2D–F). Then, we performed macrophage polarization experiments to assess the effect of MRF knockdown on macrophage polarization. Coculture with MSCs reduced the mean fluorescence intensity (MFI) of HLA-DR, and knockdown of MRF promoted a greater decrease in the MFI (Fig. 2G left panel, Fig. 2H and Additional file 4: Fig.S3A); the dot-plot analysis of M1 polarization showed similar results that coculture with MSCs reduced the ratio of CD68+/HLA-DR+M1 macrophages, knockdown of MRF strengthened decreasing tendency (Additional file 5: Fig. S4A and S4C). The levels of M1 signature genes, such as CCL5, NLRP3, and IDO1, were verified via qRT‒PCR to display similar trends (Additional file 5: Fig. S4E–S4G). The MFI of CD206 was increased upon coculture with MSCs, and knockdown of MRF further increased the MFI of CD206 (Fig. 2G right panel, Fig. 2I, and Additional file 4: Fig. S3B); the dot-plot analysis of M2 polarization revealed that the ratio of CD68+/CD206+M2 macrophages rose when coculture with MSCs, knockdown of MRF further increased the proportion of M2 macrophages (Additional file 5: Fig. S4B and S4D). The levels of the M2 signature genes CCL17, CD206, and CCL22 were detected to confirm M2 polarization (Additional file 5: Fig. S4H–S4J). Coculture with MSCs inhibited M1 macrophage polarization and promoted M2 polarization, and knockdown of MRF strengthened the regulatory effect of MSCs on macrophage polarization.

Taken together, these results indicated that MRF knockdown inhibited the recruitment of monocytes via MCP1 and enhanced the effect of MSCs on restraining M1 macrophage polarization and facilitating M2 polarization.

### Overexpression of MRF in MSCs accelerates monocyte recruitment but does not affect macrophage polarization

**Fig. 3:**
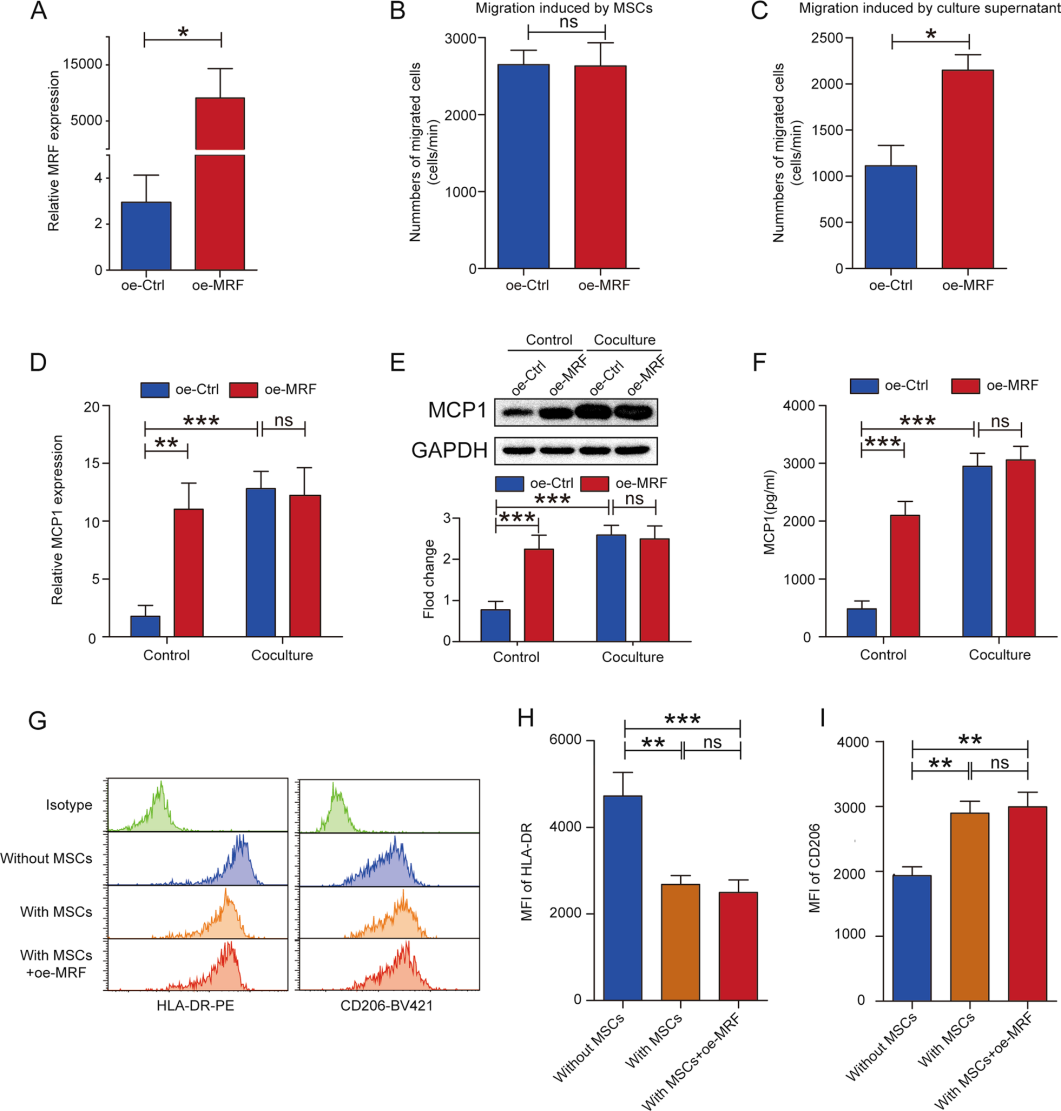
Overexpression of MRF promotes the ability of MSCs to recruit monocytes but does not affect macrophage polarization. **A** MRF overexpression was measured via qRT–PCR after lentiviral transfection (n = 3). **B** The number of migrated monocytes induced by MSCs was not significantly different after MRF overexpression (n = 3). **C** The number of migrated monocytes induced by MSC culture supernatants was increased after MRF overexpression (n = 3). **D** The MCP1 mRNA level in MSCs was measured via qRT–PCR after MRF overexpression under the conditions of coculture with or without CD14+ monocytes (n = 3). **E** The MCP1 protein level in MSCs was detected by western blotting, and quantitative results were normalized to the result for GAPDH (n = 3). **F** MCP1 levels in MSC culture supernatants were quantified via ELISA (n = 3). **G** The MFI of HLA-DR (left panel) and CD206 (right panel) was detected in CD68-positive cells via flow cytometry analysis (n = 3). **H** Histogram showing the MFI of HLA-DR among the groups cultured without MSCs, with MSCs and with MRF-overexpressing MSCs. **I** Histogram showing the MFI of CD206 among the groups cultured without MSCs, with MSCs and with MRF-overexpressing MSCs. The data are presented as the mean ± SD; *P < 0.05, **P < 0.01, ***P < 0.001, *ns* not significant. All experiments were independently repeated three times

We overexpressed MRF in MSCs via lentiviral transfection, and the overexpression efficiency was confirmed by qRT–PCR (Fig. 3A). Migration assays indicated that the MSC-induced recruitment of monocytes was not different between the overexpression and control groups (Fig. 3B). Only the MSC culture supernatant from the overexpression group attracted more monocytes than that from the control group (Fig. 3C). MCP1 expression was increased when MRF was overexpressed without monocyte coculture, but in the coculture context, MRF overexpression did not further enhance the expression level of MCP1, as detected by qRT–PCR, western blotting and ELISA (Fig. 3D–F). Macrophage polarization assays revealed that MRF overexpression in MSCs did not alter the MFI of HLA-DR (Fig. 3G left panel and Fig. 3H) or CD206 (Fig. 3G right panel and Fig. 3I) compared to the group cocultured with MSCs. The dot-plot analysis of M1 and M2 polarization showed the consistent trends that MRF overexpression did not change the ratio of CD68+/HLA-DR+ or CD68+/CD206+macrophages (Additional file 6: Fig. S5A–S5D). And the expression of M1 or M2 macrophage signature genes drew the similar conclusions (Additional file 6: Fig. S5E–S5J). Based on these results, overexpression of MRF did not further change the regulatory effect of MSCs on macrophage M1 or M2 polarization.

### MRF promotes MCP1 expression by binding to HNRNPD

**Fig. 4:**
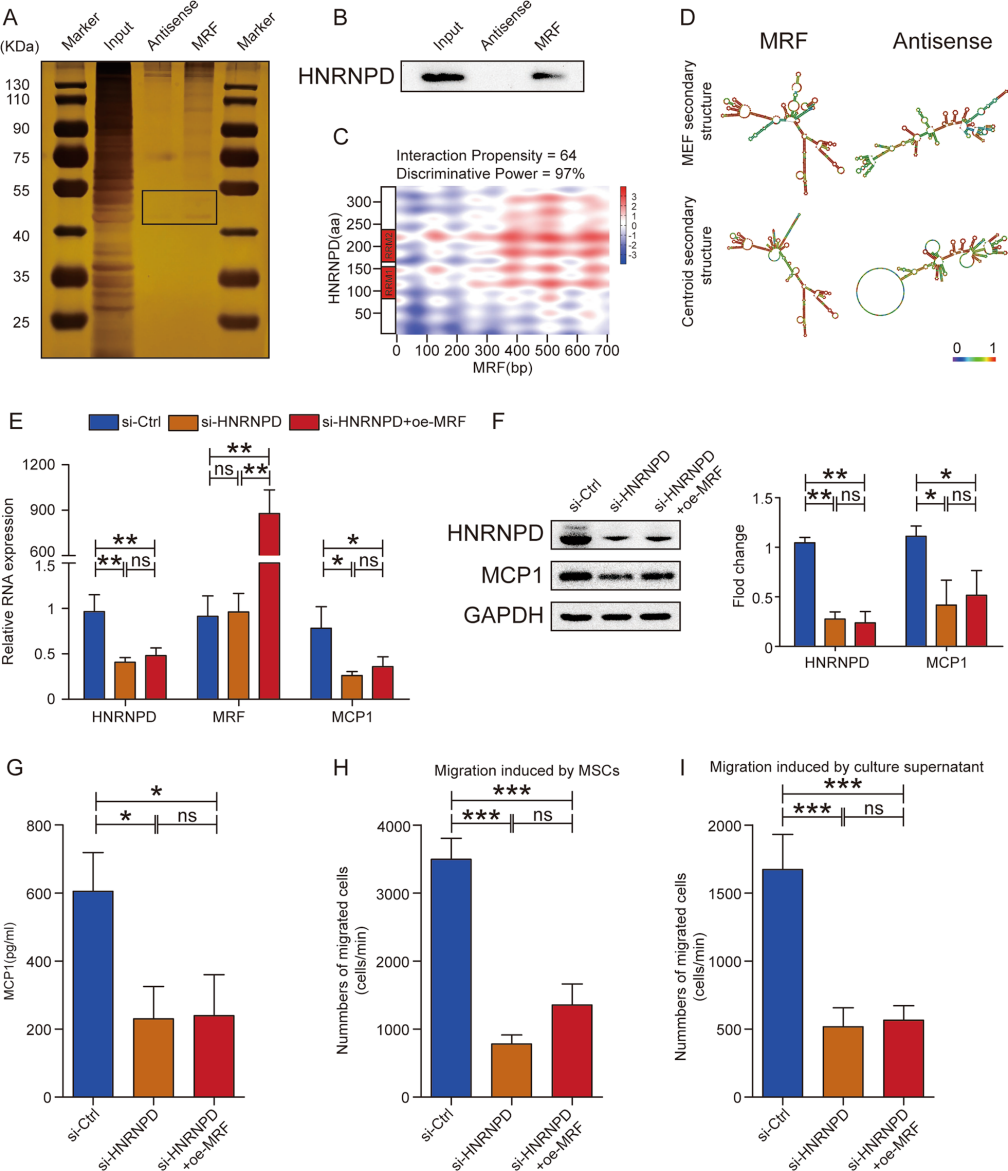
MRF binds with HNRNPD to regulate MCP1 expression. **A** Specific protein binding with MRF was identified by silver staining after RNA pull-down. **B** The interaction of MRF and HNRNPD was verified by western blotting. **C** The fragments showing binding between MRF and HNRNPD were analyzed using catRAPID. The organization of HNRNPD domains is shown on the left. **D** The predicted secondary structure of MRF was determined with the RNAfold Webserver (http://rna.tbi.ac.at/) on the basis of the minimum free energy (MFE) and partition function. **E** The mRNA levels of HNRNPD, MRF and MCP1 in MSCs were measured by qRT–PCR after HNRNPD knockdown with or without MRF overexpression (n = 3). **F** The HNRNPD and MCP1 protein levels in MSCs were detected by western blotting, and quantitative results were normalized to the result for GAPDH (n = 3). **G** MCP1 levels in MSC culture supernatants were quantified via ELISA (n = 3). **H** The number of migrated monocytes induced by MSCs was decreased after HNRNPD knockdown, and MRF overexpression did not restore the level of MCP1 (n = 3). **I** The number of migrated monocytes induced by MSC culture supernatants was decreased after HNRNPD knockdown, and MRF overexpression did not restore the level of MCP1 (n = 3). The data are presented as the mean ± SD; *P < 0.05, **P < 0.01, ***P < 0.001, *ns* not significant. All experiments were independently repeated three times

As lncRNA–protein interactions are pivotal molecular mechanisms by which lncRNAs exert their biological functions [23], we performed RNA pull-down and mass spectrometry analyses to explore downstream pathways and identify potential protein binding partners of MRF (Additional file 7: Table S2). Silver staining and western blotting results confirmed that HNRNPD specifically bound to MRF compared with the antisense sequence of MRF (Fig. 4A and B). We evaluated the propensity of the interaction between MRF and HNRNPD using an algorithm named catRAPID omics to further explore the fragment of MRF that interacted with HNRNPD. The results revealed that two RNA recognition motif domains (RRM1 and RRM2) in HNRNPD might bind to fragments covering the 350- to 700-nt region of MRF (Fig. 4C). Analysis of the secondary structure of MRF showed a stable stem–loop structure in comparison to the antisense sequence structure (Fig. 4D).

To explore the function of HNRNPD during MSC coculture with monocytes, we first detected the dynamic change in HNRNPD expression during coculture, and the results did not show a distinct trend (Additional file 8: Fig. S6A and S6B). We performed knockdown of HNRNPD in MSCs and found that MCP1 but not MRF was downregulated and that concurrent MRF overexpression did not restore the MCP1 level; qRT–PCR, western blotting and ELISA results showed similar results (Fig. 4E–G). Migration assays showed that HNRNPD knockdown in MSCs eliminated their capacity to recruit monocytes, but MRF overexpression did not restore this capacity for either MSCs or MSC culture supernatants (Fig. 4H and I). These results indicated that HNRNPD was a downstream factor of MRF and that MRF regulated MCP1 expression and monocyte recruitment by binding with HNRNPD.

### MRF upregulation is mediated by IRF1 during coculture with monocytes

**Fig. 5:**
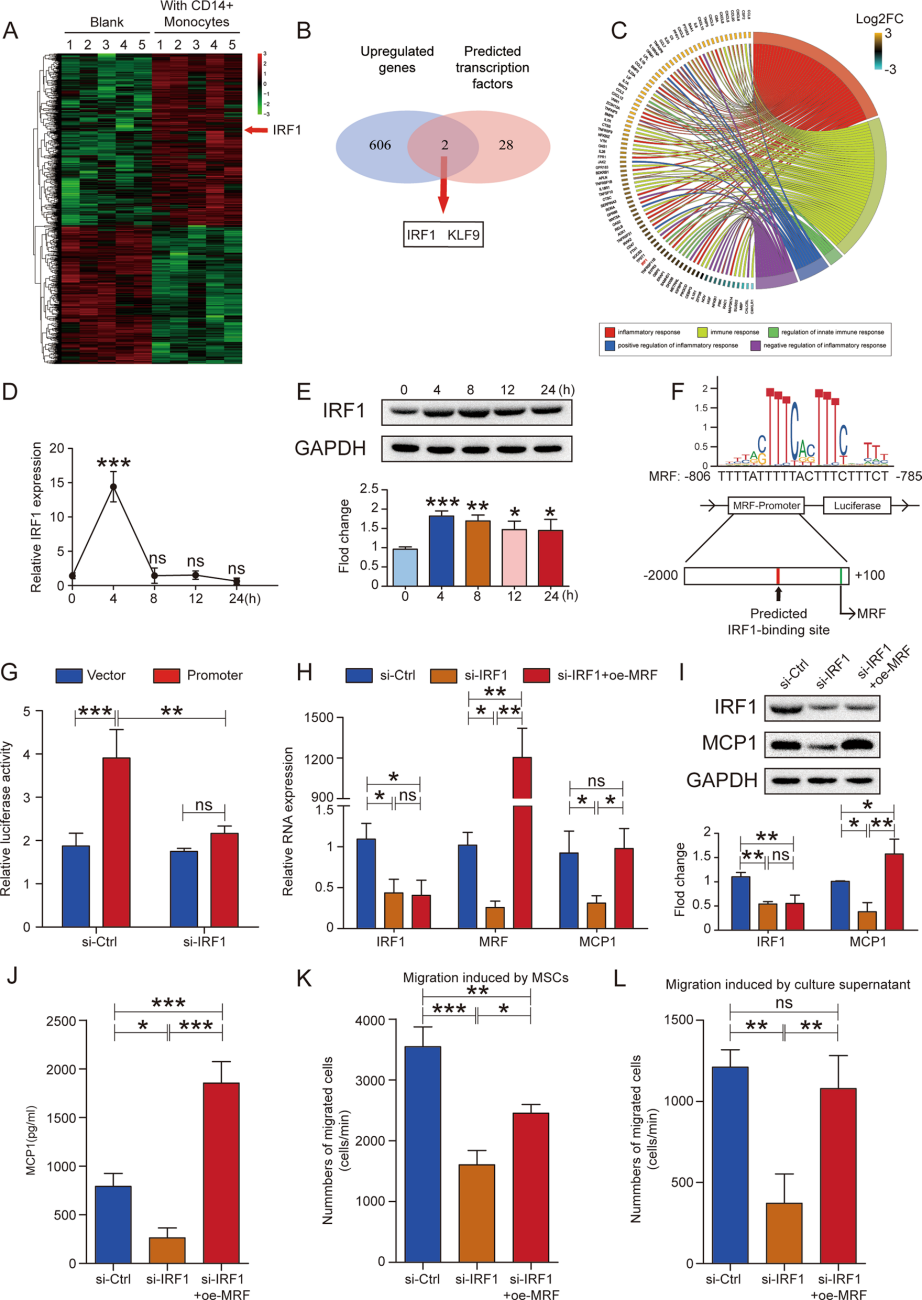
IRF1 upregulates MRF expression during coculture with monocytes. **A** Heatmap of differentially expressed mRNAs (≥ 1.5-fold) in MSCs between cells cocultured with or without CD14+ monocytes. **B** Venn diagram showing the intersection between upregulated genes and predicted transcription factors. **C** IRF1 was one of the immune-related genes identified by GOChord analysis. **D** Dynamic changes in the expression of IRF1 mRNA at different time points during coculture, as measured by qRT–PCR (n = 3). **E** IRF1 protein levels at different time points during coculture, as detected by western blotting. Quantitative results were normalized to the result for GAPDH (n = 3). **F** The sequence of the IRF1-binding site and predicted IRF1-binding site in the MRF promoter are listed in the diagram. **G** Dual-luciferase reporter assays showed that knockdown of IRF1 inhibited the luciferase activity of the reporter containing the promoter region of MRF (n = 3). **H** IRF1, MRF and MCP1 mRNA levels in MSCs were measured via qRT–PCR after IRF1 knockdown with or without MRF overexpression (n = 3). **I** IRF1 and MCP1 protein levels in MSCs were detected by western blotting, and quantitative results were normalized to the result for GAPDH (n = 3). **J** MCP1 levels in MSC culture supernatants were quantified via ELISA (n = 3). **K** The number of migrated monocytes induced by MSCs was decreased after IRF1 knockdown, and overexpression of MRF partially restored the level of MCP1 (n = 3). **L** The number of migrated monocytes induced by MSC culture supernatants was decreased after IRF1 knockdown, and overexpression of MRF partially restored the level of MCP1 (n = 3). The data are presented as the mean ± SD; *P < 0.05, **P < 0.01, ***P < 0.001, *ns* not significant. All experiments were independently repeated three times

Transcription factor-mediated regulation of gene expression is a major upstream mechanism of lncRNAs. We hypothesized that upregulated transcription factor increased MRF expression when MSCs were cocultured with monocytes. We combined the upregulated genes identified via transcriptome sequencing (Fig. 5A) with the potential transcription factors capable of binding to the MRF promoter predicted by the JASPAR database; the obtained intersection contained 2 candidates, IRF1 and KLF9 (Fig. 5B). We performed Gene Ontology (GO) functional enrichment analysis of differentially expressed genes identified by transcriptome sequencing and selected the GO terms related to the immune response to perform GOChord analysis (Fig. 5C and Additional File 9: Table S3). Eighty-seven genes, including IRF1 but not KLF9, were identified as immune-related genes, indicating that IRF1 might regulate MRF expression.

Upregulated IRF1 expression in MSCs was verified to occur during coculture with monocytes, with expression reaching a maximum at 4 h and then gradually decreasing (Fig. 5D and E). We constructed a luciferase reporter containing the MRF promoter region (Fig. 5F) and found that IRF1 knockdown reduced the luciferase activity of the reporter (Fig. 5G). Knockdown of IRF1 reduced the expression levels of MRF and MCP1, and simultaneous MRF overexpression did not change IRF1 expression but rescued MCP1 expression (Fig. 5H–J). Migration assays showed that IRF1 downregulation decreased the recruitment of monocytes induced by both MSCs and MSC culture supernatants, while MRF overexpression restored the ability to recruit monocytes to a great extent (Fig. 5K and L). These results suggested that the transcription factor IRF1 improved MRF expression early and then recruited more monocytes via MCP1 when MSCs were cocultured with monocytes.

### Downregulation of MRF in MSCs inhibits the recruitment of human monocytes and enhances MSC immunosuppressive function on macrophage polarization in a human monocyte adoptive transfer model

**Fig. 6:**
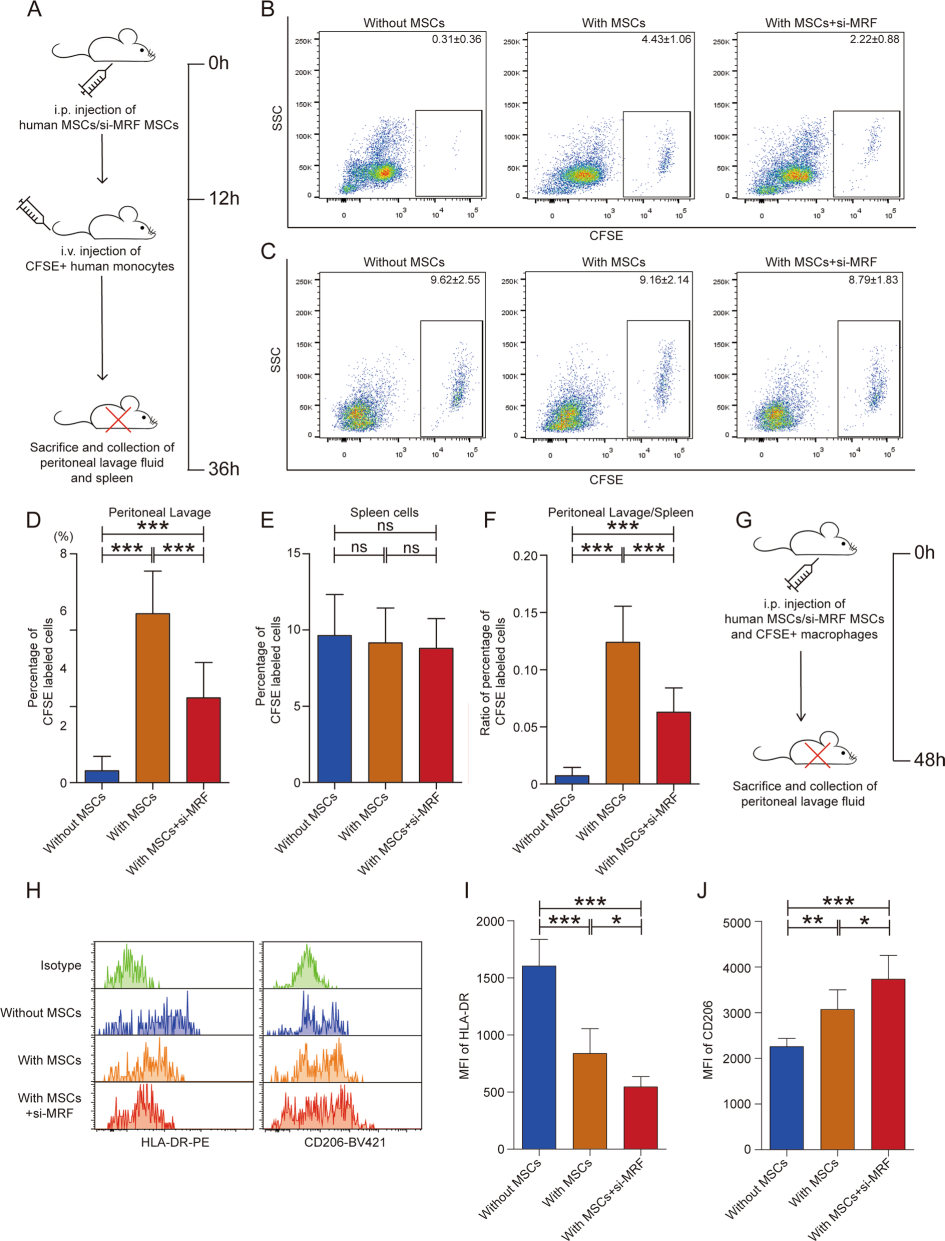
Downregulation of MRF in MSCs inhibits the recruitment of human monocytes and enhances MSC immunosuppressive function on macrophage polarization in vivo. **A** Schematic diagram of human monocyte recruitment in vivo. A human monocyte-transferred mouse model was constructed by injection of CFSE-labeled human CD14+ monocytes via the tail vein. After the injection of MSCs or si-MRF MSCs, the mice were sacrificed to collect peritoneal lavage fluid and spleen cells for flow cytometric analysis. **B** MRF-knockdown MSCs disrupted the recruitment of human monocytes into the peritoneal cavity (n = 9). **C** CFSE-labeled monocytes in the spleen cell population did not differ obviously among the groups (n = 9). **D** The proportion of CFSE-labeled monocytes in the peritoneal lavage fluid is shown as a histogram. **E** The proportion of CFSE-labeled monocytes in the spleen cell population is shown as a histogram. **F** The proportion of CFSE-labeled cells in peritoneal lavage fluid was calibrated to the percentage of CFSE-labeled monocytes in the spleen. **G** Schematic diagram of human macrophage polarization in vivo. Human MSCs/si-MRF MSCs and human macrophages labeled with CFSE were simultaneously injected intraperitoneally, and 2 days later, the mice were sacrificed to collect peritoneal lavage fluid for flow cytometric analysis. **H** The MFI of human HLA-DR (left panel) and CD206 (right panel) was detected in CFSE-labeled cells via flow cytometric analysis (n = 6). **I** Histogram showing the MFI of HLA-DR among the groups co-injected without MSCs, with MSCs and with MRF-knockdown MSCs. **J** Histogram showing the MFI of CD206 among the groups co-injected without MSCs, with MSCs and with MRF-knockdown MSCs. The data are presented as the means ± SDs; *P < 0.05, **P < 0.01, ***P < 0.001, *ns* not significant

To further verify the function of MRF in recruiting monocytes in vivo, we constructed a human monocyte-transferred mouse model by injecting carboxyfluorescein diacetate *N*-succinimidyl ester (CFSE)-labeled human CD14+ monocytes into NOD/SCID mice via the tail vein, and these mice were intraperitoneally injected with human MSCs pretreated with MRF siRNA or negative control. Peritoneal lavage fluid and spleen cells were collected for flow cytometry analysis (Fig. 6A). Compared with intraperitoneal injection of saline alone, intraperitoneal injection of human MSCs recruited CFSE-labeled human monocytes to the abdominal cavity. Furthermore, interfering with MRF expression in MSCs disrupted the recruitment of monocytes (Fig. 6B, D and Additional file 10: Fig. S7A). The proportion of CFSE-labeled monocytes among total spleen cells reflected the real quantity of human monocytes in mice, and analysis of spleen cells showed that there were no obvious differences among the groups (Fig. 6C, E and Additional file 10: Fig. S7B). To eliminate interference caused by individual differences in mice, we further calibrated the ratio of CFSE-labeled cells in peritoneal lavage fluid according to the percentage of CFSE-labeled cells in the spleen (Fig. 6F) and obtained results similar to those described above.

In addition, we assessed the role of MRF in macrophage polarization in vivo. Human MSCs were pretreated with MRF siRNA or negative control, and human macrophages were activated and stimulated to induce M1 or M2 polarization and labeled with CFSE. Then, the MSCs and macrophages were injected into the abdominal cavity of NOD/SCID mice. Two days later, peritoneal lavage fluid was collected for flow cytometry analysis (Fig. 6G). The results were consistent with the results of the in vitro experiments, showing that in the presence of MSCs, the MFI of HLA-DR was reduced (Fig. 6H left panel, Fig. 6I and Additional file 11: Fig. S8A) and that of CD206 was increased in CFSE-labeled cells (Fig. 6H right panel, Fig. 6J and Additional file 11: Fig. S8B). Knockdown of MRF intensified the trend, supporting the idea that MSCs exerted anti-inflammatory functions to inhibit M1 macrophage polarization and promote M2 polarization and that knockdown of MRF enhanced the anti-inflammatory effect of MSCs.

Overall, the in vivo experiments indicated that MRF downregulation in MSCs inhibited monocyte recruitment and enhanced the immunosuppressive effects of MSCs to inhibit M1 polarization and promote M2 polarization.

## Discussion

In the present study, we identified a new lncRNA termed MRF, which was upregulated early in MSCs during coculture with CD14+ monocytes. MRF was involved in the proinflammatory regulatory function of MSCs to accelerate monocyte recruitment, and MRF knockdown enhanced the regulatory effect of MSCs on restraining M1 polarization and facilitating M2 polarization. Further studies revealed that MRF bound to HNRNPD to increase MCP1 expression and that MRF was upregulated by the transcription factor IRF1. Animal experiments produced consistent results, showing that MRF in MSCs can recruit more monocytes.

MSCs are adult stem cells with a great immunomodulatory ability and are widely applied in clinical treatment [4, 24]. Recent studies have shown that MSCs exhibit bidirectional immunomodulation capacities under different circumstances, which are dependent on the various inflammatory statuses [2, 5, 6]. (1) When the immune system is not yet activated, MSCs can mobilize immune cells by secreting kinds of cytokines, including MCP1, and exhibit proinflammatory features [2, 5, 25]. (2) When the immune system is activated, MSCs will transform into an immunosuppressive state. Sufficient TNFα and IFN-γ concentrations trigger the anti-inflammatory phenotype of MSCs by producing substantial amounts of iNOS/IDO [26]. IL-17, a classical proinflammatory cytokine, can further enhance the immunosuppressive capacity of MSCs induced by TNFα and IFN-γ [27]. Proinflammatory cytokines secreted by M1 macrophages can activate the anti-inflammatory regulation function of MSCs to induce repolarization of M1 macrophages to M2 macrophages by upregulating the expression of COX2 and IDO [3]. The bidirectional immunomodulation of MSCs is dependent on the inflammatory status of the microenvironment and is vital to maintain the immune system in a normal state and avoid overactivation.

However, in some pathological states, MSC-mediated immunomodulation is dysfunctional, which exacerbates the disease [28, 29]. Feng et al. found that bone marrow MSCs from patients with RA expressed relatively little A20, which responded to TNFα and suppressed inflammation [30]. Loss of A20 in RA-MSCs disrupted the balance of T helper 17 (Th17)/T regulatory (Treg) cells, resulting in arthritis progression. Toro et al. showed that bone marrow Nestin + MSCs directed inflammatory monocyte migration and aortic inflammatory infiltration and accelerated atherosclerosis progression through MCP1 production [31]. Previously, we drew a similar conclusion, noting that MSCs from patients with AS expressed relatively high amounts of MCP1 and recruited excess monocytes to lesions during osteogenesis, leading to hyperactive inflammation [13]. However, the specific mechanisms by which MSCs secrete MCP1 and affect the regulation of monocytes/macrophages are still unclear.

LncRNAs are noncoding RNA transcripts that are involved in many cellular biological functions. Recently, many studies have focused on determining the roles of lncRNAs in the trilineage differentiation of MSCs [16, 17]. However, few studies have explored the relationship between lncRNAs and MSC-mediated immunomodulation [32]. Zhao et al. reported that lncRNA MALAT1 enhanced the immunosuppressive properties of MSCs by promoting polarization of THP-1 cells toward the M2 phenotype via IDO [33].

To elucidate the mechanisms by which MSCs initiate proinflammatory regulation and illustrate the processes by which MSCs secrete MCP1 and exert immunomodulatory effects on monocytes/macrophages, we previously performed high-throughput sequencing of lncRNA expression profiles for the first time by comparing untreated MSCs with MSCs cocultured with CD14+ monocytes, and 145 lncRNAs were identified as differentially expressed genes [12]. In the present study, we further screened an immune-related lncRNA termed MRF that was upregulated early in MSCs during coculture with CD14+ monocytes.

Knockdown and overexpression experiments confirmed that MRF was vital to maintain the ability of MSCs to recruit monocytes via MCP1. Knockdown of MRF enhanced the effect of MSCs on restraining M1 macrophage polarization and facilitating M2 polarization. Based on these results, MRF exhibited proinflammatory characteristics and was involved in MSC-mediated immunomodulation.

To date, many studies have focused on the immunomodulatory effects of MSCs under the circumstances of activated immune system and have confirmed the conclusions on the anti-inflammatory function of MSCs [34–36]; however, this study aimed to explore how MSCs initiated an inflammatory response when the immune system was not activated. At coculture initiation, which lacked adequate inflammatory signals, MRF was upregulated in MSCs to promote monocyte recruitment and macrophage polarization. With the accumulation of immune cells, MRF expression gradually decreased. Notably, the results of the macrophage polarization assay in the present study were consistent with the results of previous studies; coculture with MSCs inhibited M1 macrophage polarization and facilitated M2 polarization compared to the group without coculture with MSCs [26, 37, 38]. The main difference was that knockdown of MRF alone enhanced the effect of MSCs on macrophage polarization, confirming the proinflammatory characteristic of MRF. In summary, the conclusions of this study are not contradictory but complementary to those of previous studies, supporting the view that MSC-mediated immunomodulation is bidirectional.

Interestingly, when MRF was overexpressed, only the group that was induced by the MSC culture supernatant, not the group directly induced by MSCs, exhibited increased monocyte recruitment, and similar results were obtained in the macrophage polarization assay. The MSC culture supernatant group did not receive direct stimulation by monocytes, but the direct MSC induction group did. We hypothesized that endogenous MRF was upregulated when MSCs were cocultured with monocytes and subsequently initiated the inflammatory response and that additional MRF overexpression could not further activate the inflammatory pathways. Chen et al. revealed that overexpression of the lncRNA LNMAT1 in bladder cancer cells induced excess MCP1 expression and recruited tumor-associated macrophages (TAMs) [39], resulting in lymphatic metastasis. It was obvious that bladder cancer cells had no ability to maintain an immunomodulatory balance similar to MSCs, and overexpression of this proinflammatory lncRNA further activated the immune response regardless of the original level of inflammation.

Heterogeneous nuclear ribonucleoproteins (hnRNPs) are a family of RNA-binding proteins (RBPs) that influence mRNA metabolism through different pathways and participate in many biological functions [40, 41]. Here, we found that MRF interacted with HNRNPD to exert specific effects. Recently, many studies have shown that HNRNPD acts as an effector molecule of lncRNAs [42]. Ainara et al. reported that lnc13 bound to HNRNPD to regulate the expression of inflammatory genes by changing the epigenetic state of chromatin, affecting susceptibility to celiac disease [43]. lncRNA AFAP1-AS1 interacts with HNRNPD, promotes the translation of ERBB2, and induces drug resistance in breast cancer [44]. Li et al. reported that LINC01354 interacted with HNRNPD to stabilize the CTNNB1 mRNA transcript, thereby activating the Wnt/β-catenin signaling pathway in colorectal cancer [45]. Our results showed that HNRNPD downregulation decreased MCP1 expression but not MRF expression and inhibited monocyte recruitment. In contrast, overexpression of MRF did not rescue MCP1 expression or the impaired recruitment capacity. These data indicated for the first time that HNRNPD positively regulated MCP1 as a downstream protein of MRF. Here, we hypothesized that HNRNPD can stabilize the MCP1 mRNA transcript by binding to the 3′ untranslated region (UTR) of the MCP1 mRNA transcript and that MRF enhances this effect by promoting the interaction between HNRNPD and MCP1 mRNA. The detailed process by which the MRF-HNRNPD-MCP1 axis works remains to be further studied.

Transcription factor-mediated gene transcription is a classic pathway for lncRNA expression regulation. Our data confirmed that the transcription factor IRF1 acted as an upstream factor to activate MRF transcription and initiate the proinflammatory regulatory activity of MSCs. Kim et al. showed that IRF1 was involved in the TLR4-mediated proinflammatory response of MSCs [46]; this result was consistent with our current findings. IRF1 mainly activates the expression of type I interferon genes to regulate innate immunity and host antiviral defenses [47]. Feng et al. showed that viral infection induced IRF1 transcriptional activation in the early phase and that the IRF1 level subsequently decreased, exhibiting rapid and dynamic expression regulation [48]. Short-lived IRF1 maintained a proinflammatory state for pathogen defense and avoided overactivation of the immune system during the later stage. Our study showed a similar tendency for IRF1 when MSCs were cocultured with CD14+ monocytes, reflecting the dynamic variation in MSC-mediated immunomodulation, which maintained homeostasis of the immune microenvironment.

A human monocyte adoptive transfer mouse model is suitable for studying the chemotaxis and infiltration of human monocytes in vivo. An adoptive transfer model was constructed by intravenously injecting human CD14+ peripheral monocytes into nude mice along with a subcutaneous injection of recombinant human M-CSF [20]. Human monocytes could be detected in the circulation and were recruited to the lungs, spleen and orthotopic pulmonary metastases. Sidibe et al. performed adoptive transfer of human monocytes into NOD/SCID mice and found that human monocytes were recruited to the abdominal cavity in a nontumoral inflammatory peritonitis model [21]. In our study, intraperitoneal injection of human MSCs induced a proinflammatory state to attract monocytes, whereas pretreatment of MSCs to induce MRF knockdown decreased the recruitment of monocytes to the abdominal cavity, supporting the hypothesis that MRF in MSCs regulated monocyte chemotaxis. We also performed human macrophage polarization in vivo via co-injection of human MSCs and macrophages into the abdominal cavity, and the results were consistent with those of in vitro experiments, showing that knockdown of MRF enhanced the immunosuppressive effects of MSCs to inhibit M1 macrophage polarization and promote M2 polarization.

There are still some unresolved questions in this study. How do MSCs recognize monocyte signals at the beginning of coculture and initiate the proinflammatory process via MRF? What is the specific mechanism by which MRF binds to HNRNPD to regulate MCP1 expression? Is MRF expression abnormal, and is it part of the pathogenesis of MSC-related immunomodulatory dysfunction in autoimmune diseases? Further studies on MRF need to be conducted. MRF is a potential target for genetic modification to enhance the immunomodulatory ability of MSCs in clinical applications, and MRF may become a therapeutic target to specifically influence pathological MSCs in certain diseases.

## Conclusions

In conclusion, we identified a new lncRNA termed MRF in MSCs that regulated monocyte recruitment and polarization through the HNRNPD-MCP1 axis. MRF was upregulated when MSCs were cocultured with monocytes and increased MCP1 expression to accelerate monocyte recruitment and regulated macrophage polarization to the M1 rather than the M2 phenotype. Knockdown of MRF inhibited monocyte recruitment, enhanced the regulatory effect of MSCs on restraining M1 polarization and facilitating M2 polarization. Mechanistically, MRF bound with HNRNPD to increase MCP1 expression and was upregulated by IRF1 in the early coculture period. We propose that the IRF1/MRF/HNRNPD/MCP1 axis is involved in the immunoregulatory ability of MSCs. MRF may become a key target to improve the clinical effect of MSC-based therapy or correct MSC-related immunomodulatory dysfunction under pathological states.

## Supplementary Information

**Additional file 1: Table S1.** List of primers.

**Additional file 2: Fig. S1.** Protein-coding potential analysis and conservation analysis of MRF. (A) MRF exhibited little protein-coding potential, as analyzed using CPAT, CPC and PhyloCSF. (B) The conservation analysis of MRF was conducted with PhyloP and PhastCons.

**Additional file 3: Fig. S2.** MCP1 expression in MSCs during coculture with monocytes. (A-C) Dynamic changes of MCP1 expression in MSCs at different time points during coculture, as measured by qRT–PCR, western blotting and ELISA. (D-F) The knockdown efficiency of MCP1 in monocytes was verified via qRT–PCR, western blotting and ELISA. (G–I) Dynamic changes of MCP1 expression in MSCs at different time points during coculture upon knockdown of MCP1 in monocytes, as detected by qRT–PCR, western blotting and ELISA. Quantitative results of western blotting were normalized to the result for GAPDH. The data are presented as the mean ± SD; *p < 0.05, **p < 0.01, ***p < 0.001, ns= not significant. n=3, all experiments were independently repeated three times.

**Additional file 4: Fig. S3.** The gating strategy for determination of in vitro human macrophage polarization via flow cytometry analysis. (A) The gating strategy for identification of M1 macrophage polarization in vitro. (B) The gating strategy for identification of M2 macrophage polarization in vitro.

**Additional file 5: Fig. S4. **The dot-plot analysis and the expression of signature genes of macrophage polarization upon MRF knockdown. (A) The dot-plots of M1 macrophage polarization. (B) The dot-plots of M2 macrophage polarization. (C) The ratio of CD68+/HLA-DR+ cells. (D) The ratio of CD68+/CD206+ cells. (E-G) The expression of the M1 signature genes CCL5, NLRP3 and IDO1 was measured via qRT‒PCR. (H-J) The expression of the M2 signature genes CCL17, CD206 and CCL22 was measured via qRT‒PCR. The data are presented as the mean ± SD; *p < 0.05, **p < 0.01, ***p < 0.001, ****p < 0.0001, ns= not significant. n=3, all experiments were independently repeated three times.

**Additional file 6: Fig. S5.** The dot-plot analysis and the expression of signature genes of macrophage polarization upon MRF overexpression. (A) The dot-plots of M1 macrophage polarization. (B) The dot-plots of M2 macrophage polarization. (C) The ratio of CD68+/HLA-DR+ cells. (D) The ratio of CD68+/CD206+ cells. (E-G) The expression of the M1 signature genes CCL5, NLRP3 and IDO1 was detected via qRT‒PCR. (H-J) The expression of the M2 signature genes CCL17, CD206 and CCL22 was detected via qRT‒PCR. The data are presented as the mean ± SD; *p < 0.05, **p < 0.01, ***p < 0.001, ns= not significant. n=3, All experiments were independently repeated three times.

**Additional file 7: Table S2.** HNRNPD bound to MRF, as detected by mass spectrometry.

**Additional file 8: Fig. S6. **HNRNPD expression in MSCs during coculture with CD14+ monocytes. (A) Dynamic changes in HNRNPD mRNA expression at different time points during coculture, as measured by qRT–PCR (n=3). (B) The HNRNPD protein level at different time points during coculture was detected by western blotting. Quantitative results were normalized to the result for GAPDH (n=3). The data are presented as the mean ± SD; ns= not significant. All experiments were independently repeated three times.

**Additional file 9: Table S3.** IRF1 was identified as a transcription factor that regulated MRF expression.

**Additional file 10: Fig. S7.** The gating strategy for assessment of in vivo human monocyte recruitment via flow cytometry analysis. (A) The gating strategy for human CFSE-labeled monocytes in peritoneal lavage fluid. (B) The gating strategy for identification of human CFSE-labeled monocytes among spleen cells.

**Additional file 11: Fig. S8.** The gating strategy for determination of in vivo human macrophage polarization via flow cytometry analysis. (A) The gating strategy for determination of in vivo M1 macrophage polarization. (B) The gating strategy for determination of in vivo M2 macrophage polarization.

## Data Availability

The datasets used and/or analyzed in the current study are available from the corresponding author on reasonable request.

## Abbreviations

MSCs: Mesenchymal stem cells
lncRNAs: Long noncoding RNAs
MRF: LncRNA MCP1 regulatory factor
MCP1: Monocyte chemotactic protein 1
CCL2: CC-chemokine ligand 2
HNRNPD: Heterogeneous nuclear ribonucleoprotein D
IRF1: Interferon regulatory factor 1
AS: Ankylosing spondylitis
PBMCs: Peripheral blood mononuclear cells
RACE: Rapid amplification of cDNA ends
siRNAs: Small interfering RNAs
ELISA: Enzyme-linked immunosorbent assay
GO: Gene ontology
MFI: Mean fluorescence intensity
CFSE: Carboxyfluorescein diacetate N-succinimidyl ester
