# Supplementary material for: LncRNA MRF drives the regulatory function on monocyte recruitment and polarization through HNRNPD-MCP1 axis in mesenchymal stem cells
Source: J Biomed Sci. 2022 Sep 21;29:73. doi: 10.1186/s12929-022-00858-3 (PMC9490984; doi:10.1186/s12929-022-00858-3)
Supplement: Supplementary file 1 — Additional file 1: Table S1. List of primers. [file 12929_2022_858_MOESM1_ESM.docx]

**Table S1**. List of primers.

| Primer | Sequence (5’-3’) |
| --- | --- |
| 3'RACE-GSP | GATTACGCCAAGCTTGGCTGGGAGAGAAAGCCTCCGTGA |
| 3'RACE-NGSP | GATTACGCCAAGCTTTGCCGATTCTGCCAGCAAATCCATC |
| 5'RACE-GSP | GATTACGCCAAGCTTAGGGTAACATGAGTGGTCGGGAAACA |
| 5'RACE-NGSP | GATTACGCCAAGCTTAGCTTGCACTCTGGCACTCACACA |
| MRF-F | TGCCCACTACCTCATTCCCA |
| MRF-R | TGGAGTGGAGACTGTTCCGT |
| U6-F | CTCGCTTCGGCAGCACA |
| U6-R | AACGCTTCACGAATTTGCGT |
| MALAT1-F | GTCATAACCAGCCTGGCAGT |
| MALAT1-R | CGAAACATTGGCACACAGCA |
| GAPDH-F | GGAGCGAGATCCCTCCAAAAT |
| GAPDH-R | GGCTGTTGTCATACTTCTCATGG |
| ACTB-F | CATGTACGTTGCTATCCAGGC |
| ACTB-R | CTCCTTAATGTCACGCACGAT |
| MCP1-F | CAGCCAGATGCAATCAATGCC |
| MCP1-R | TGGAATCCTGAACCCACTTCT |
| HNRNPD-F | GCGTGGGTTCTGCTTTATTACC |
| HNRNPD-R | TTGCTGATATTGTTCCTTCGACA |
| IRF1-F | CTGTGCGAGTGTACCGGATG |
| IRF1-R | ATCCCCACATGACTTCCTCTT |
| NLRP3-F | GATCTTCGCTGCGATCAACAG |
| NLRP3-R | CGTGCATTATCTGAACCCCAC |
| CCL5-F | CCAGCAGTCGTCTTTGTCAC |
| CCL5-R | CTCTGGGTTGGCACACACTT |
| IDO1-F | GCCAGCTTCGAGAAAGAGTTG |
| IDO1-R | ATCCCAGAACTAGACGTGCAA |
| CCL17-F | CTCCAGGGATGCCATCGTTT |
| CCL17-R | TCTCTTGTTGTTGGGGTCCG |
| CD206-F | GGGTTGCTATCACTCTCTATGC |
| CD206-R | TTTCTTGTCTGTTGCCGTAGTT |
| CCL22-F | ATCGCCTACAGACTGCACTC |
| CCL22-R | GACGGTAACGGACGTAATCAC |
| MCP1-F | CAGCCAGATGCAATCAATGCC |
| MCP1-R | TGGAATCCTGAACCCACTTCT |
