# Supplementary figures and images for: LncRNA MRF drives the regulatory function on monocyte recruitment and polarization through HNRNPD-MCP1 axis in mesenchymal stem cells
Source: J Biomed Sci. 2022 Sep 21;29:73. doi: 10.1186/s12929-022-00858-3 (PMC9490984; doi:10.1186/s12929-022-00858-3)

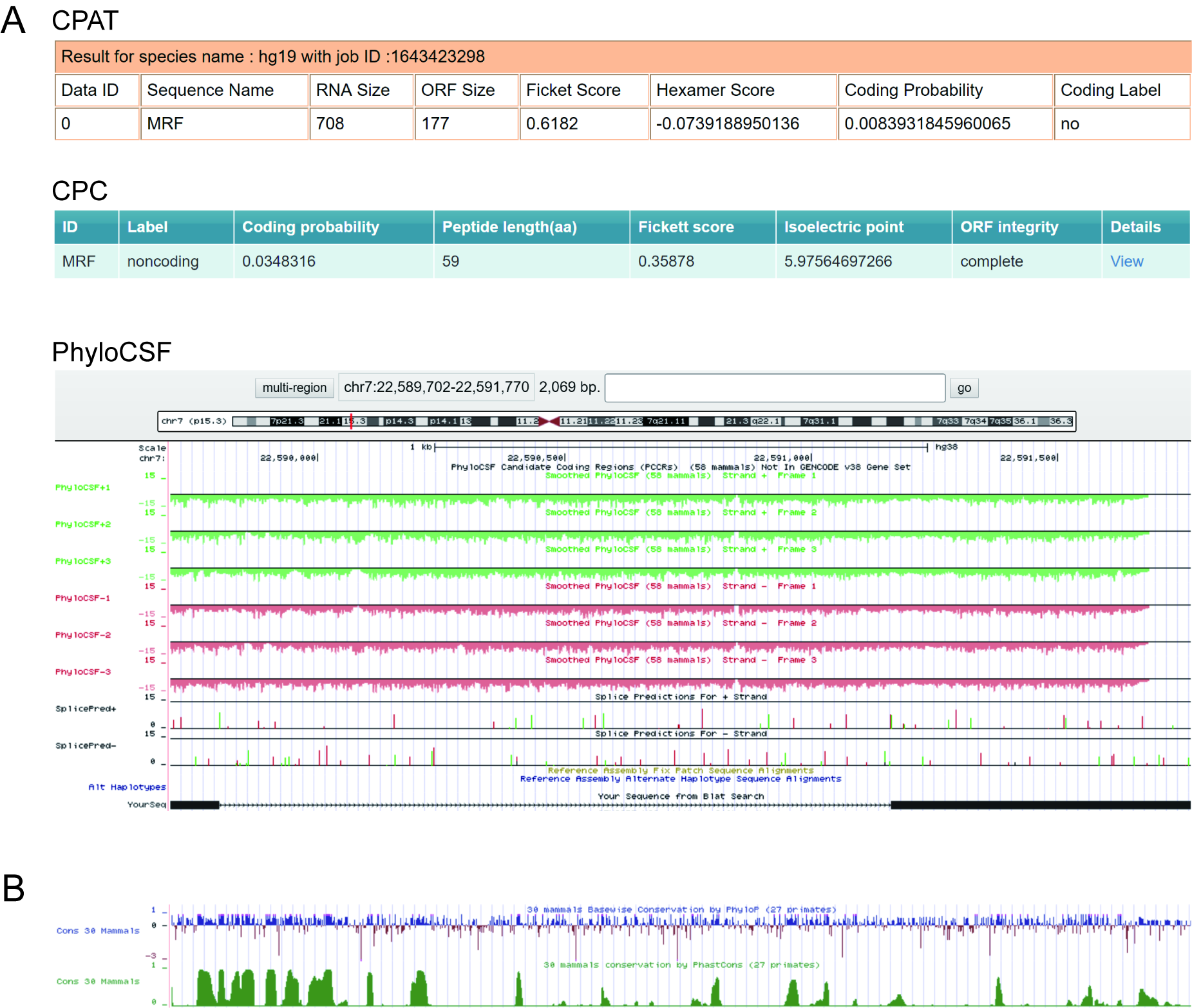

Supplement: Supplementary file 2 — Additional file 2: Fig. S1. Protein-coding potential analysis and conservation analysis of MRF. (A) MRF exhibited little protein-coding potential, as analyzed using CPAT, CPC and PhyloCSF. (B) The conservation analysis of MRF was conducted with PhyloP and PhastCons. [file 12929_2022_858_MOESM2_ESM.tif]

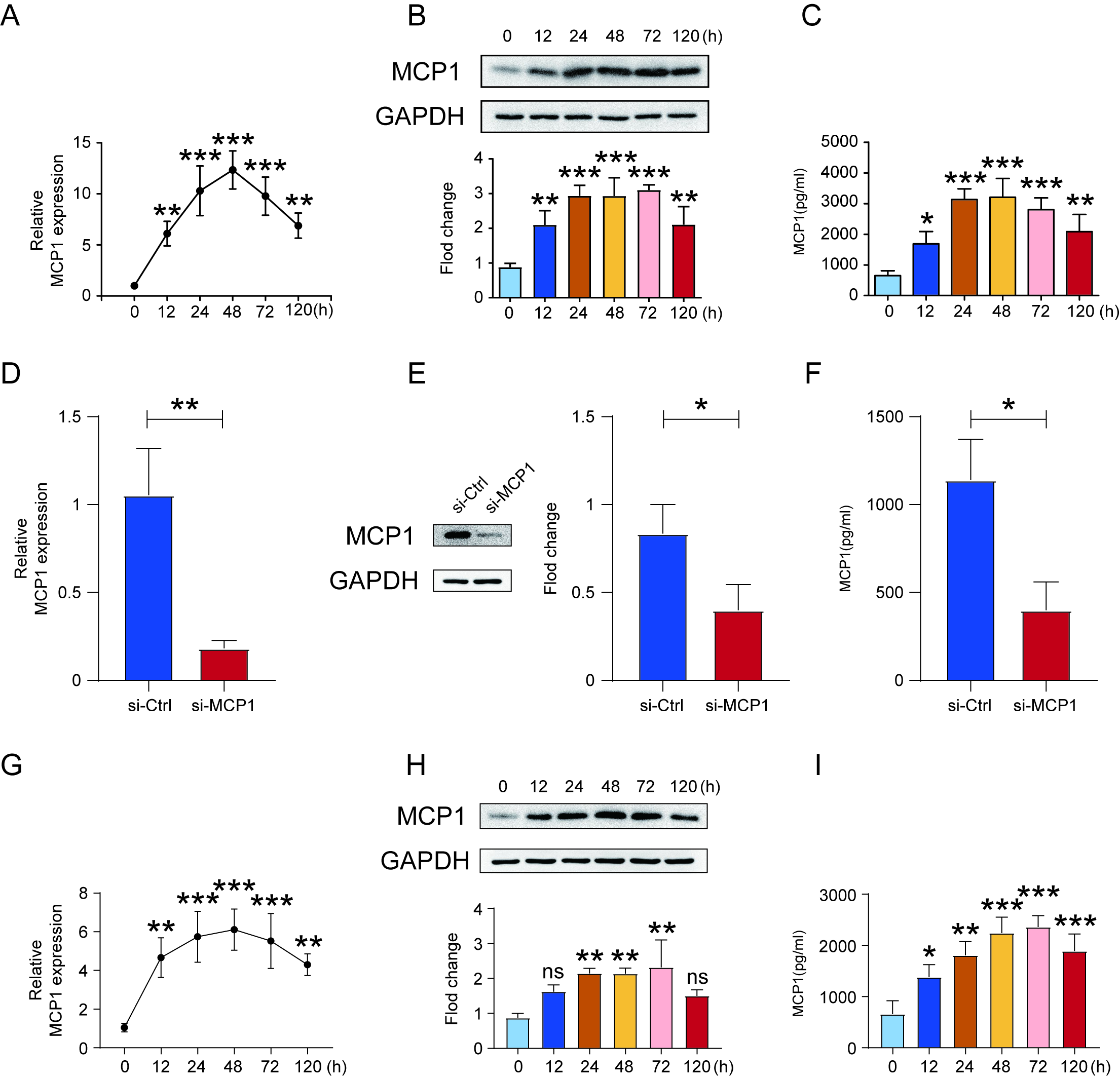

Supplement: Supplementary file 3 — Additional file 3: Fig. S2. MCP1 expression in MSCs during coculture with monocytes. (A-C) Dynamic changes of MCP1 expression in MSCs at different time points during coculture, as measured by qRT–PCR, western blotting and ELISA. (D-F) The knockdown efficiency of MCP1 in monocytes was verified via qRT–PCR, western blotting and ELISA. (G–I) Dynamic changes of MCP1 expression in MSCs at different time points during coculture upon knockdown of MCP1 in monocytes, as detected by qRT–PCR, western blotting and ELISA. Quantitative results of western blotting were normalized to the result for GAPDH. The data are presented as the mean ± SD; *p < 0.05, **p < 0.01, ***p < 0.001, ns= not significant. n=3, all experiments were independently repeated three times. [file 12929_2022_858_MOESM3_ESM.tif]

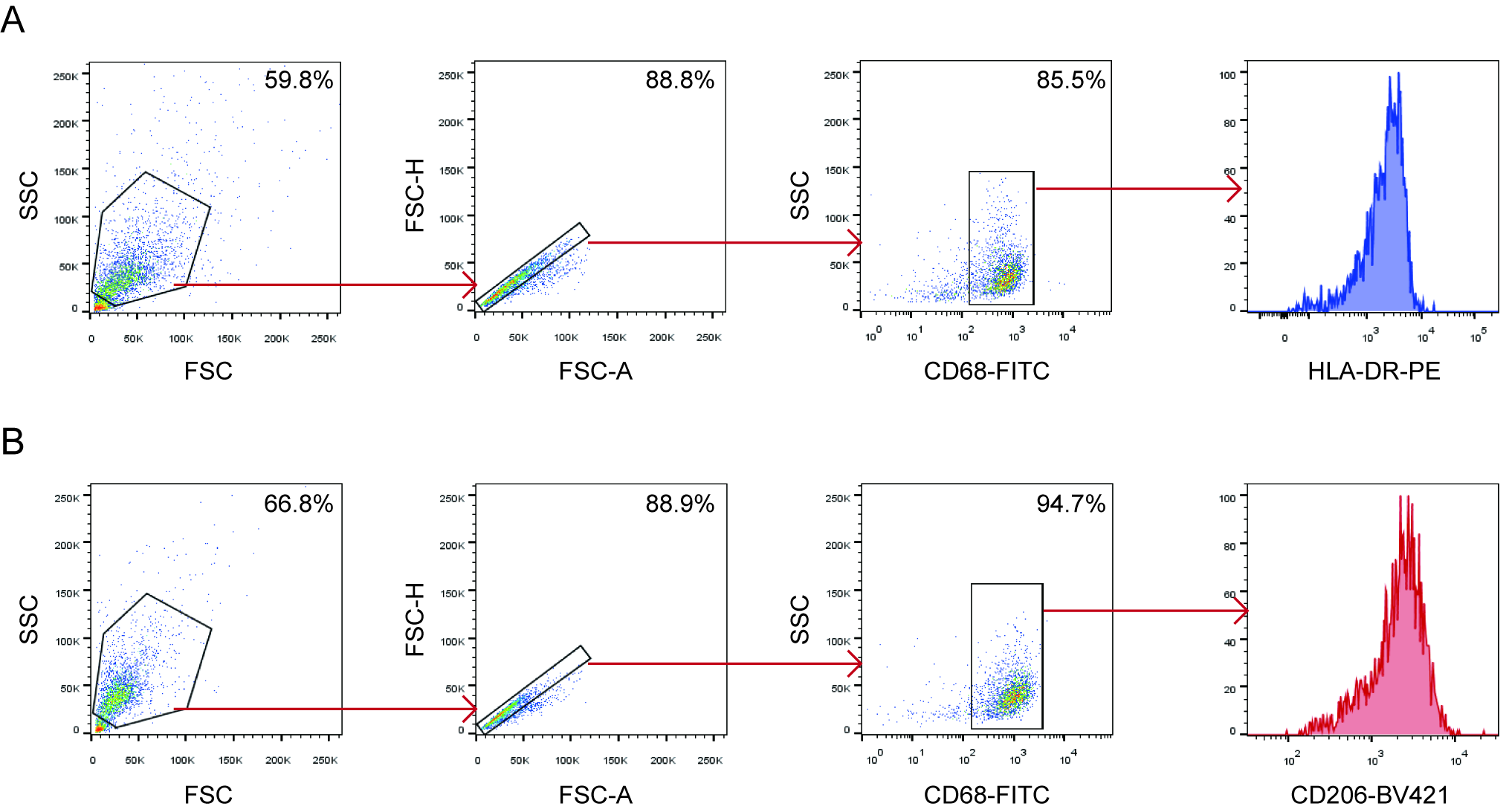

Supplement: Supplementary file 4 — Additional file 4: Fig. S3. The gating strategy for determination of in vitro human macrophage polarization via flow cytometry analysis. (A) The gating strategy for identification of M1 macrophage polarization in vitro. (B) The gating strategy for identification of M2 macrophage polarization in vitro. [file 12929_2022_858_MOESM4_ESM.tif]

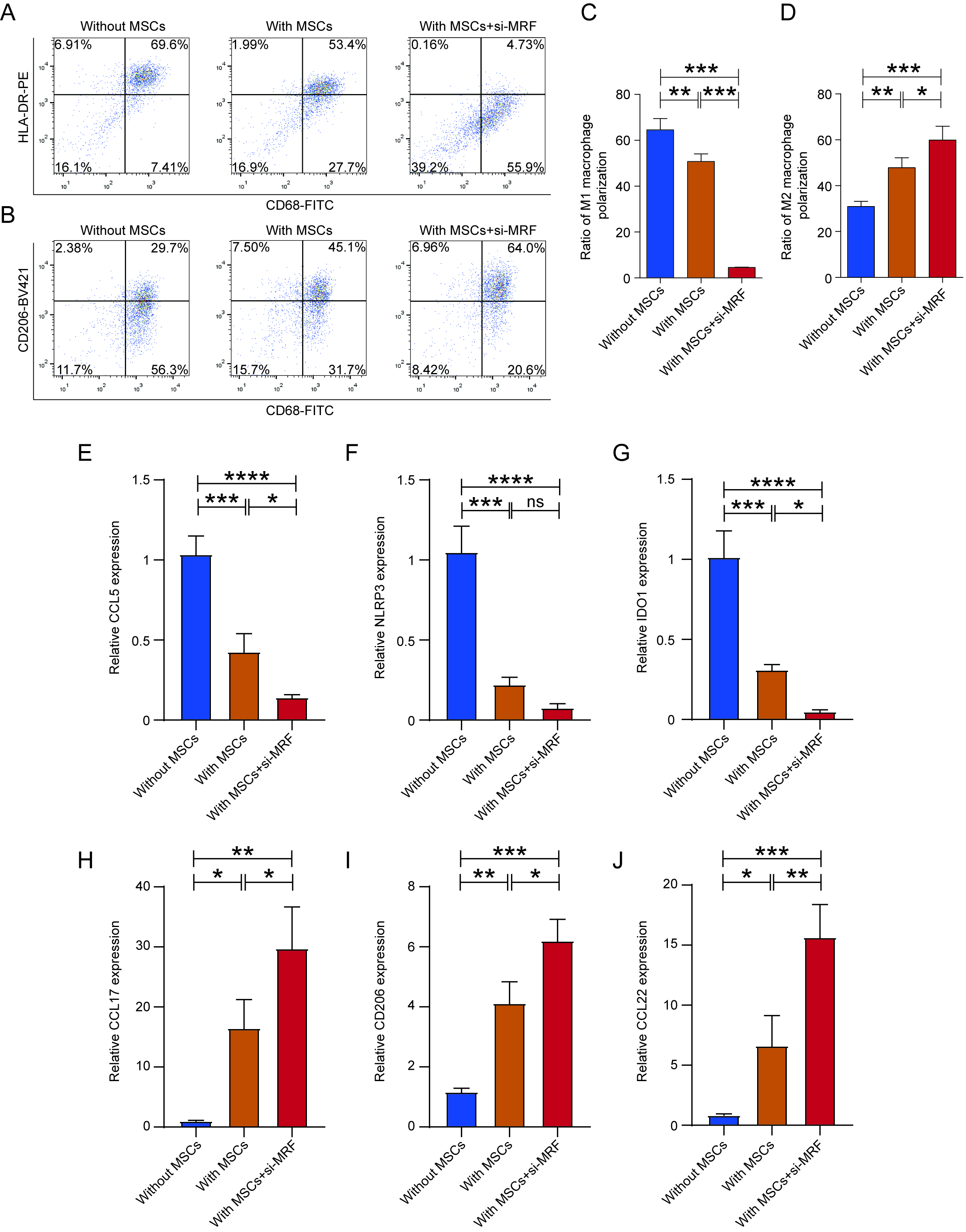

Supplement: Supplementary file 5 — Additional file 5: Fig. S4. The dot-plot analysis and the expression of signature genes of macrophage polarization upon MRF knockdown. (A) The dot-plots of M1 macrophage polarization. (B) The dot-plots of M2 macrophage polarization. (C) The ratio of CD68+/HLA-DR+ cells. (D) The ratio of CD68+/CD206+ cells. (E-G) The expression of the M1 signature genes CCL5, NLRP3 and IDO1 was measured via qRT‒PCR. (H-J) The expression of the M2 signature genes CCL17, CD206 and CCL22 was measured via qRT‒PCR. The data are presented as the mean ± SD; *p < 0.05, **p < 0.01, ***p < 0.001, ****p < 0.0001, ns= not significant. n=3, all experiments were independently repeated three times. [file 12929_2022_858_MOESM5_ESM.tif]

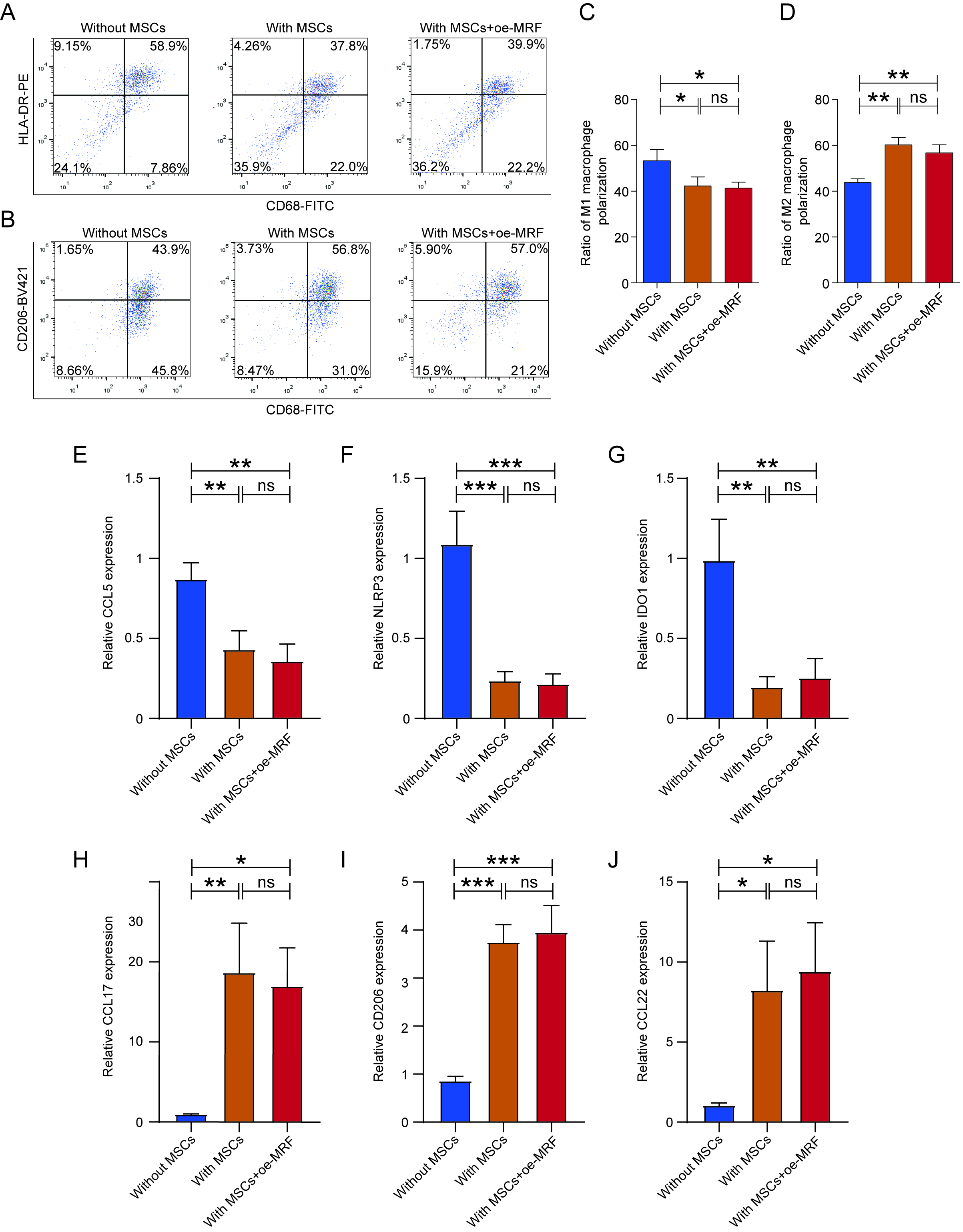

Supplement: Supplementary file 6 — Additional file 6: Fig. S5. The dot-plot analysis and the expression of signature genes of macrophage polarization upon MRF overexpression. (A) The dot-plots of M1 macrophage polarization. (B) The dot-plots of M2 macrophage polarization. (C) The ratio of CD68+/HLA-DR+ cells. (D) The ratio of CD68+/CD206+ cells. (E-G) The expression of the M1 signature genes CCL5, NLRP3 and IDO1 was detected via qRT‒PCR. (H-J) The expression of the M2 signature genes CCL17, CD206 and CCL22 was detected via qRT‒PCR. The data are presented as the mean ± SD; *p < 0.05, **p < 0.01, ***p < 0.001, ns= not significant. n=3, All experiments were independently repeated three times. [file 12929_2022_858_MOESM6_ESM.tif]

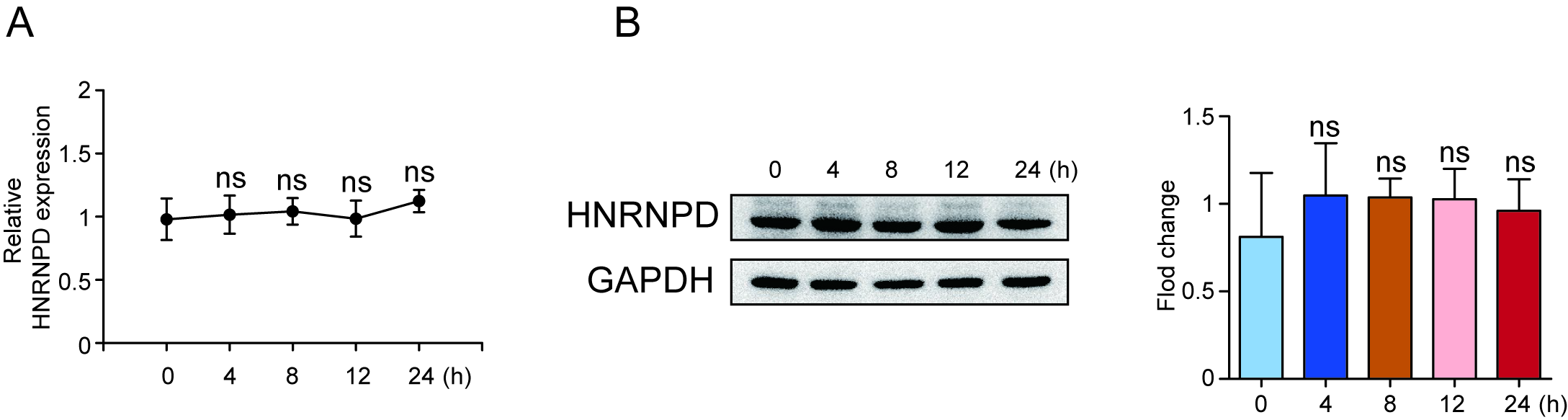

Supplement: Supplementary file 8 — Additional file 8: Fig. S6. HNRNPD expression in MSCs during coculture with CD14+ monocytes. (A) Dynamic changes in HNRNPD mRNA expression at different time points during coculture, as measured by qRT–PCR (n=3). (B) The HNRNPD protein level at different time points during coculture was detected by western blotting. Quantitative results were normalized to the result for GAPDH (n=3). The data are presented as the mean ± SD; ns= not significant. All experiments were independently repeated three times. [file 12929_2022_858_MOESM8_ESM.tif]

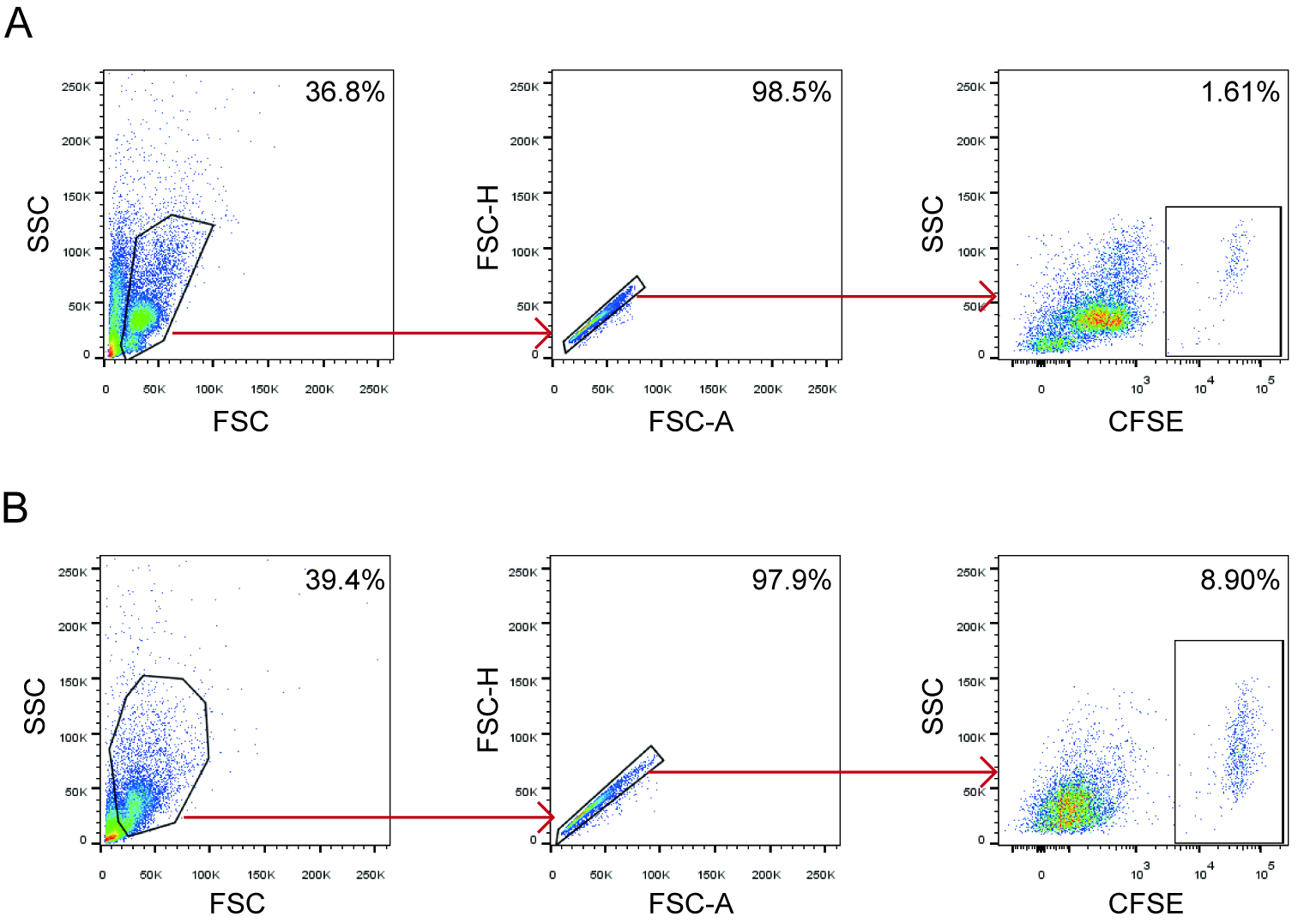

Supplement: Supplementary file 10 — Additional file 10: Fig. S7. The gating strategy for assessment of in vivo human monocyte recruitment via flow cytometry analysis. (A) The gating strategy for human CFSE-labeled monocytes in peritoneal lavage fluid. (B) The gating strategy for identification of human CFSE-labeled monocytes among spleen cells. [file 12929_2022_858_MOESM10_ESM.tif]

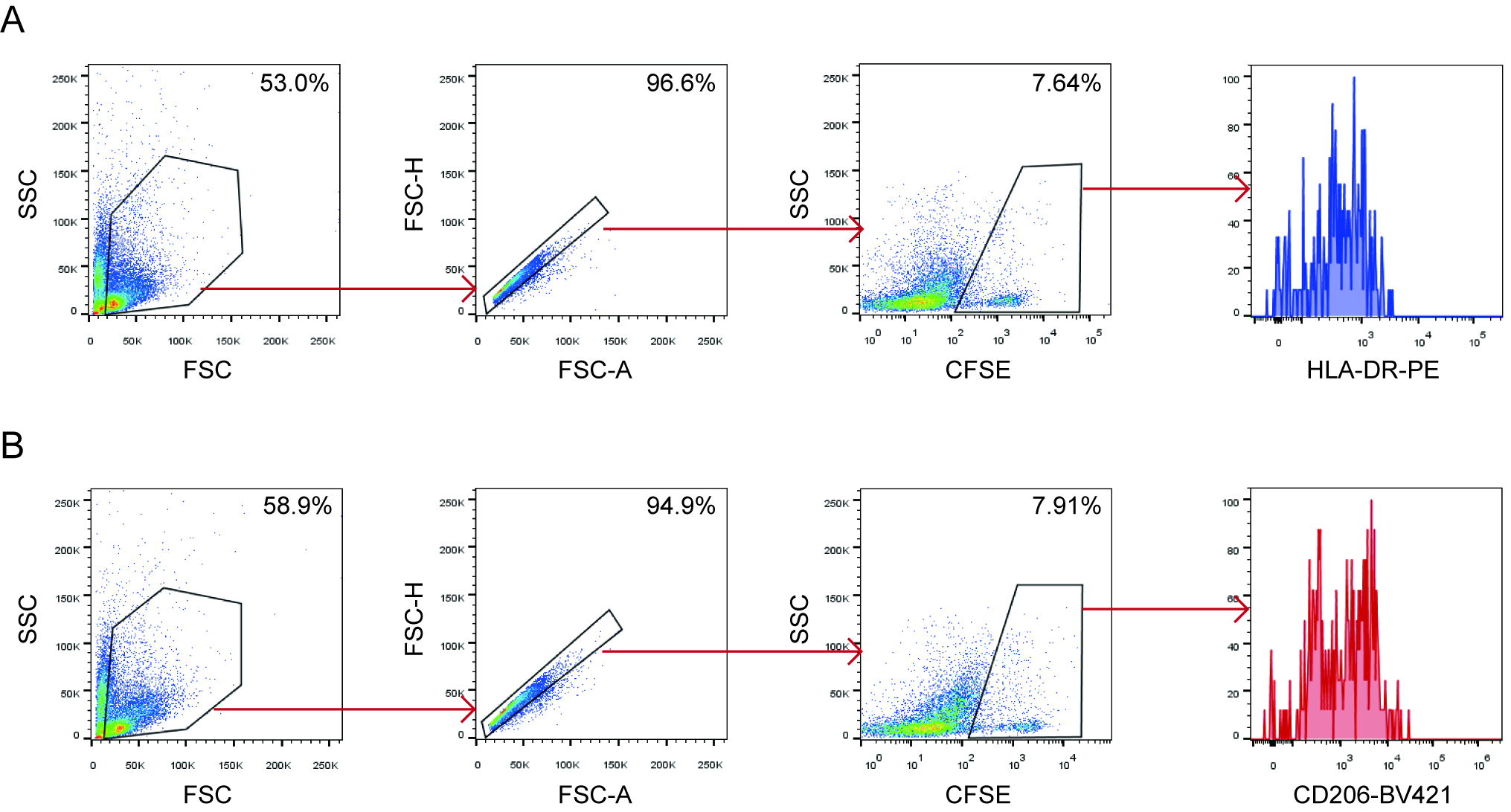

Supplement: Supplementary file 11 — Additional file 11: Fig. S8. The gating strategy for determination of in vivo human macrophage polarization via flow cytometry analysis. (A) The gating strategy for determination of in vivo M1 macrophage polarization. (B) The gating strategy for determination of in vivo M2 macrophage polarization. [file 12929_2022_858_MOESM11_ESM.tif]
